# Discrete μ_4_‑Oxido Mn(II) Clusters with Controlled Nuclearity: On-Demand Synthesis, Molecular and Self-Assembled Structures

**DOI:** 10.1021/acsomega.5c06133

**Published:** 2025-09-26

**Authors:** Dominik Jabłoński, Maciej Jacyna, Michał Terlecki, Arkadiusz Kornowicz, Iwona Justyniak, Janusz Lewiński

**Affiliations:** † Faculty of Chemistry, 49566 Warsaw University of Technology, Noakowskiego 3, 00-664 Warsaw, Poland; ‡ Institute of Physical Chemistry, 49559 Polish Academy of Sciences, Kasprzaka 44/52, 01-224 Warsaw, Poland

## Abstract

Discrete heterovalent manganese-oxido clusters are widely used as building blocks and precursors of modern functional materials, such as molecular magnets, polyoxometalates (POMs), metal-oxide nanocrystals, and metal–organic frameworks (MOFs). While access to various heterovalent Mn­(II)/Mn­(III) oxido clusters is well established, the rational synthesis of similar compounds composed exclusively of Mn­(II) centers remains a significant challenge. Herein, we present an on-demand synthesis of homovalent [Mn_4_^II^(μ_4_-O)­L_6_]-type clusters via a transmetalation/hydrolysis approach. Interestingly, by tuning the coordination properties of selected model organic ligands, we achieved control over the nuclearity of the resulting manganese oxide clusters, leading to the formation of either tetranuclear [Mn_4_(μ_4_-O)­(L^NN^)_6_] or octanuclear [Mn_4_(μ_4_-O)­(L^ON^)_6_]_2_ clusters, depending on the use of *N*,*N*′- or *O*,*N*-bidentate ligands, respectively. Furthermore, a detailed analysis of the supramolecular structures of these new complexes, in comparison with selected examples of widely studied zinc-oxido clusters and to a lesser extent with Co­(II) and Fe­(II) analogues, provided deeper insight into the factors controlling solvation and noncovalent interaction-driven self-assembly.

## Graphical abstract

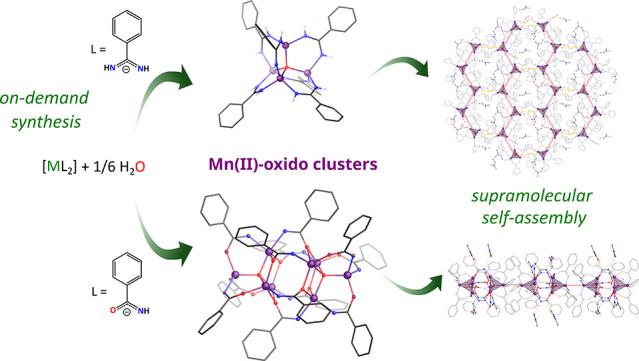

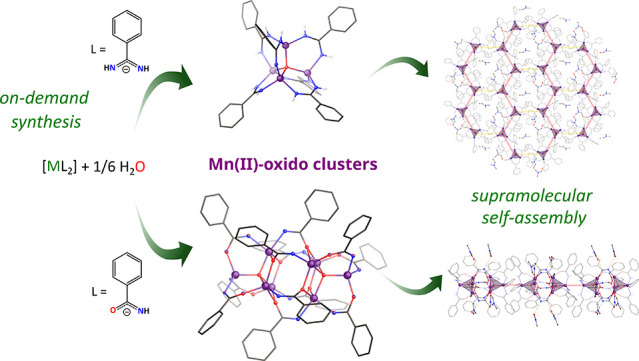

## Introduction

Metal-oxido clusters offer a diverse landscape of functional molecular moieties due to their structural diversity, physicochemical properties, and rich chemistry. To date, polyoxometalates (POMs) have been extensively exploited as catalysts^1,2^ and building blocks of desired materials;^3–7^ however, the application of lower nuclearity clusters remains a highly underexplored area. Discrete O-centered metal-oxido clusters incorporating organic ligand shells can be utilized as building blocks or precursors for the fabrication of more complex functional systems,^8,9^ including polyoxometalate (POM) nanoclusters,^10,11^ metal-oxide nanocrystals,^12–16^ and porous metal–organic frameworks (MOFs).^17–21^ Moreover, these small molecular clusters can be regarded as soluble representations of fundamental structural units of higher-nuclearity POMs and the crystal lattices of metal oxides. As such, they can serve as a valuable predesigned platform for investigating various aspects of metal-oxide chemistry, like unveiling intimate steps in (co)­polymerization of epoxides,^22^ expanding knowledge on the factors governing phase transitions and polymorph-controlled syntheses,^23^ modeling MOFs’ reactivity toward water and small donor molecules,^24^ mapping pathways in MOFs^25^ and metal–organic gel assembly,^26^ and searching the growth mechanism of nanocrystals^13,15^ and the coordination dynamics of ligands on their surface.^15,27^ More recently, molecular metal clusters have emerged as promising building units for noncovalent functional supramolecular frameworks, such as the noncovalent porous materials (NPMs)^28–30^ or molecular porous assemblies,^31–33^ and metal hydrogen-bonded organic frameworks,^34,35^ which are easily processable^30,34^ and may exhibit interesting flexible behavior^23,30^ or highly selective gas adsorption.^30^ These frameworks are organized via intermolecular noncovalent interactions governed by the secondary coordination sphere (SCS), which remains challenging to control.^36^ Harnessing the SCS in combination with intermolecular noncovalent interactions provides an additional level of tailorability of metal-oxido clusters and their deliberate utilization as well-defined building blocks of functional materials. To advance these applications, it is essential to facilitate access to molecular metal-oxide clusters with tailored characteristics and deepen the understanding of their chemical behavior, including the interplay between the primary and secondary coordination spheres.

One of the most ubiquitous building units of metal-oxido structures is the tetrahedral oxido-centered [M_4_(μ_4_-O)]^n+^ species.^37^ This structural motif is particularly widespread in zinc coordination chemistry, where discrete clusters with a general formula of [Zn_4_(μ_4_-O)­L_6_] form an extended family of hybrid organic–inorganic compounds incorporating a vast array of stabilizing ligands (L) with various steric and electronic characteristics.^38,39^ Such diversity in structural features results in highly tunable optoelectronic,^40–42^ coordination,^19,24,43–45^ and self-assembly properties,^23,29,36,39,46^ which turn these μ_4_-oxido metal clusters into suitable candidates for luminescent materials,^40,41,47^ catalysts,^48–51^ and precursors of isoreticular MOFs.^20,52^ In turn, the versatility of ligands that can be incorporated into [Zn_4_(μ_4_-O)­L_6_]-type clusters makes them ideal platforms for a systematic study of how controlled modifications in the SCS affect their noncovalent interaction-driven self-assembly. For example, simple zinc-oxido carboxylates and amidinates incorporating various substituents in the aromatic backbone in the distal SCS can form diverse types of noncovalent assemblies ranging from structures representing zeolitic topologies to soft porous materials with gated voids or open channels.^29,39^ On the other hand, we demonstrated that the NH groups in the proximal SCS are prone to the formation of intermolecular hydrogen bonds, which can be used to tailor the packing of [Zn_4_(μ_4_-O)­L_6_]-type clusters toward NPMs in the crystal structure.^36,53^ While research on the physicochemical properties of molecular metal-oxido clusters has been extensively conducted for decades, the crystal engineering of noncovalent frameworks involving this family of metal clusters remains a challenging and highly undeveloped field. Noncovalent interactions between molecules give rise to molecular recognition and self-assembly processes, and often play a dominant role in the synthesis, catalysis, and design of materials.^54–58^ For example, adjusting both the character of the metal center along with the secondary coordination sphere in building-block clusters can provide a promising means to control the properties of the resulting materials.^59^ Thus, in-depth understanding of these interactions is crucial across various fields of chemistry and materials science.^60^

Strikingly, while the facile access to [M_4_(μ_4_-O)­L_6_]-type complexes based on Zn­(II) ions is provided by well-established synthetic procedures,^39,61^ the rational preparation of similar oxido clusters based on open-shell transition metal ions, like Fe­(II), Co­(II), or Mn­(II), is still highly challenging, which significantly hampers the development of [M_4_(μ_4_-O)]-based materials.^20,45^ Only very recently, we have successfully introduced a novel efficient procedure, called the transmetalation/hydrolysis approach, for the efficient preparation of homometallic [M_4_(μ_4_-O)­L_6_]-type clusters based not only on Zn­(II) but also other divalent transition metal ions.^53^ On this week, we demonstrated the first on-demand synthesis of new isostructural Fe­(II) and Co­(II) amidato clusters [M_4_(μ_4_-O)­(L^ON^)_6_] (M = Co, Fe; H-L^ON^ = benzamide) as well as the known Zn­(II) analogue. Now, we extended the application of this approach to the rational synthesis of new Mn­(II)-oxido clusters, tetranuclear [Mn_4_(μ_4_-O)­(L^NN^)_6_] (**1-Mn**) and octanuclear [Mn_4_(μ_4_-O)­(L^ON^)_6_]_2_ (**2**_**2**_) stabilized by *N*,*N*′-bidentate benzamidinato (L^NN^) and *O*,*N*-bidentate benzamidato (L^ON^) ligands, respectively.

Multinuclear manganese-oxido clusters are of particular interest due to their structural tunability^62,63^ and intriguing magnetic and redox properties.^64^ For instance, a dodecanuclear cluster [Mn_12_O_12_(OAc)_16_(H_2_O)_4_] has emerged as a prototypical single-molecule magnet,^65^ paving the way for the development of the field of molecular magnetism, and currently, various manganese-oxido species serve as building units of modern magnetic materials.^64,66,67^ In turn, the CaMn_4_O_*x*_ cluster constitutes the active site of photosystem II (PSII), catalyzing the water oxidation reaction and, by mimicking its mode of action, various manganese-oxido clusters demonstrate promising catalytic activity in oxidation processes.^68,69^ More recently, manganese-oxido clusters have also been used as promising precursors for the fabrication of manganese oxide nanocrystals.^70^

The [Mn_4_(μ_4_-O)]^n+^ species are typical building units of heterovalent Mn­(II)/Mn­(III) manganese-oxido clusters with higher nuclearity, which readily form during the oxidation of Mn­(II) carboxylates or inorganic salts. In these reactions, the oxidation agent, usually KMnO_4_ or O_2_ from the air, acts as the source of the O^2–^ anions, simultaneously increasing the oxidation state of some metal centers. For instance, [Mn_4_^II^Mn_2_^III^O_2_(L)_10_(X)_n_]-type clusters (L = e.g., RCOO, X = neutral ligands, e.g., RCOOH, H_2_O, pyridine) bearing {Mn_4_^II^Mn_2_^III^(μ_4_-O)}^10+^ core composed of two edge-sharing {Mn_2_^II^Mn_2_^III^O}^8+^ tetrahedra (Figure 1c) are common products of the oxidation of simple Mn­(II) carboxylates.^71–77^ In turn, introducing various multidentate stabilizing ligands into the reaction system resulted in manganese-oxido clusters with higher nuclearity and a variety of core structures. The examples include a series of decanuclear [Mn_4_^II^Mn_6_^III^O_4_(L)_*y*_(X)_*x*_]^z+^-type clusters (X = N_3_, Br, or I) comprising supertetrahedral cores built from four vertex-sharing [Mn^II^Mn_3_^III^O]^9+^ tetrahedra stabilized by various tripodal alcoholates (Figure 1d),^78–81^ a dodecanuclear cluster [Mn_4_^II^Mn_8_^III^O_4_(H_2_O)_2_(Cl)_8_(*edte*)_4_] (*edte*H_4_ **=*N*,*N*,*N*′,*N*′-tetrakis­(2-hydroxyethyl)­ethylenediamine) with a twisted saddle-like core built from four vertex-sharing [Mn^II^Mn_3_^III^O]^9+^ tetrahedra (Figure 1e),^82^ and a highly symmetrical tridecanuclear cluster [Mn^II^Mn_12_^III^O_8_(Cl)_6_(O_3_P^t^Bu)_8_] comprising a cuboctahedral core composed of eight edge-fused [Mn^II^Mn_3_^III^O]^9+^ tetrahedra sharing one centrally located Mn^II^ vertex (Figure 1f).^83^ Contrary to partially oxidized heterovalent manganese-oxido clusters, the rational synthesis of homovalent Mn­(II)-oxido clusters is still challenging due to the high reactivity of the Mn­(II) species. To our knowledge, there are only two reports on the isolation of discrete [Mn_4_^II^(μ_4_-O)­L_6_]-type clusters, both incorporating monoanionic amidinato ligands (*N*,*N*′-diphenylformamidine^84,85^ and *N*,*N*′-dimethylformamidine^86^ anions). Moreover, Wright et al. reported on the isolation and structural characterization of a dimeric octanuclear cluster [Mn_4_^II^(μ_4_-O)­L_6_]_2_ (LH = 2-amino-3-bromo-5-methyl-pyridine), in which two [Mn_4_^II^(μ_4_-O)]^6+^ units are connected by bridging organic *N*,*N*′-ligands.^87^ Strikingly, all of these Mn­(II)-oxido clusters were obtained serendipitously due to the water contamination of the reactants or the reaction system.

**Figure 1:**
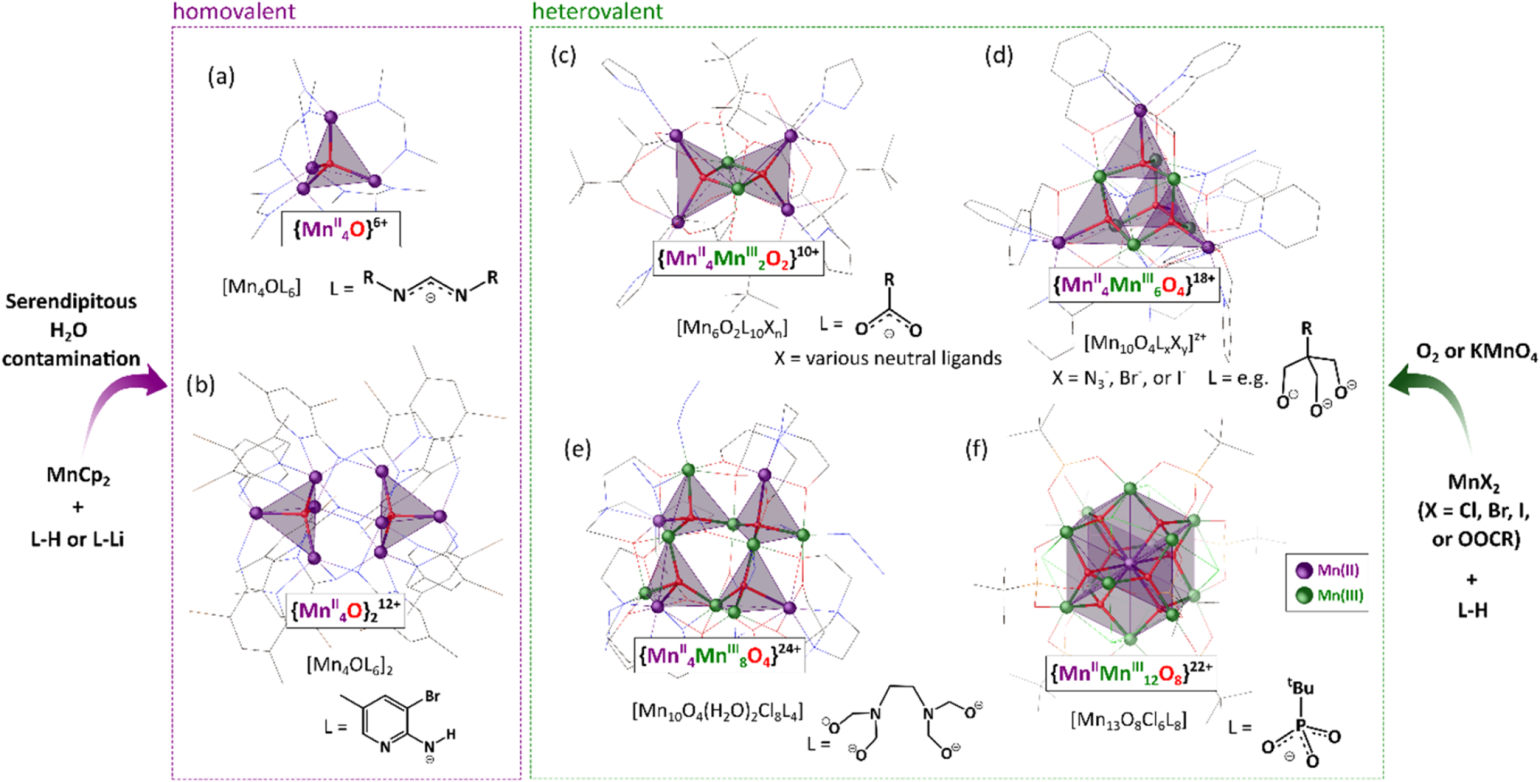
Previously isolated homovalent manganese-oxido [Mn_4_^II^(μ_4_-O)]^6+^-based clusters (a, b) and selected examples of heterovalent manganese-oxido clusters composed of [Mn_4_^II/III^(μ_4_-O)]^n+^ building units (c–f).

Herein, we present the first synthetic approach enabling the on-demand preparation of homovalent [Mn_4_(μ_4_-O)­(L^NN^)_6_]-type oxido clusters. We demonstrated its effectiveness by preparing two new clusters, tetranuclear [Mn_4_(μ_4_-O)­(L^NN^)_6_] (**1-Mn**) and octanuclear [Mn_4_(μ_4_-O)­(L^ON^)_6_]_2_ (**2**_**2**_) stabilized by *N*,*N*′-bidentate benzamidinato (L^NN^) and *O*,*N*-bidentate benzamidato (L^ON^) ligands, respectively. The selected model ligands exhibit distinct coordination properties, enabling control over the nuclearity of the resulting manganese-oxido clusters. Furthermore, they introduce varying numbers of hydrogen-bond donor and acceptor sites into the proximal secondary coordination sphere of the clusters, which plays a substantial role in directing their self-assembly.^36^

## Results and Discussion

### Transmetalation/Hydrolysis Approach for Mn­(II)-Oxido Clusters

Applying the previously designed one-pot three-step transmetalation/hydrolysis approach^53^ two new homovalent Mn­(II)-oxido clusters, tetranuclear [Mn_4_^II^(μ_4_-O)­(L^NN^)_6_] (**1-Mn**) and octanuclear [Mn_4_^II^(μ_4_-O)­(L^ON^)_6_]_2_ (**2**_**2**_), were obtained. The synthetic procedure involves (i) the generation of potassium salt from the selected proligand L-H via the reaction with KH, (ii) the transmetalation reaction with MnCl_2_, and (iii) the stoichiometric hydrolysis of the received homoleptic [Mn^II^(L)_2_]_*x*_-type derivative (Figure 2). The respective Mn­(II)-oxido products were obtained in the form of well-defined yellowish crystals after extraction with the mixture of DMF/THF (1:3, v/v) and subsequent crystallization upon slow diffusion of hexane vapor. Notably, while cluster **1-Mn** was isolated with a high yield (73%) through a single extraction process, in the case of cluster **2**_**2**_, due to its low solubility, the extraction had to be repeated three times to obtain a satisfactory yield of 69% (see the Experimental Section). The phase purity of isolated products was confirmed by powder X-ray diffraction (PXRD) analysis (Figure S11). Both Mn­(II) complexes were highly sensitive toward O_2_ and turned brown immediately under slight exposure to air. Clusters **1-Mn** and **2**_**2**_ were characterized by single-crystal X-ray diffraction (SC-XRD), ^1^H NMR and FTIR spectroscopy, and elemental analysis (for details, see the SI; note that due to the low solubility of **2**_**2**_, ^1^H NMR analysis was performed only for **1-Mn**).

**Figure 2:**
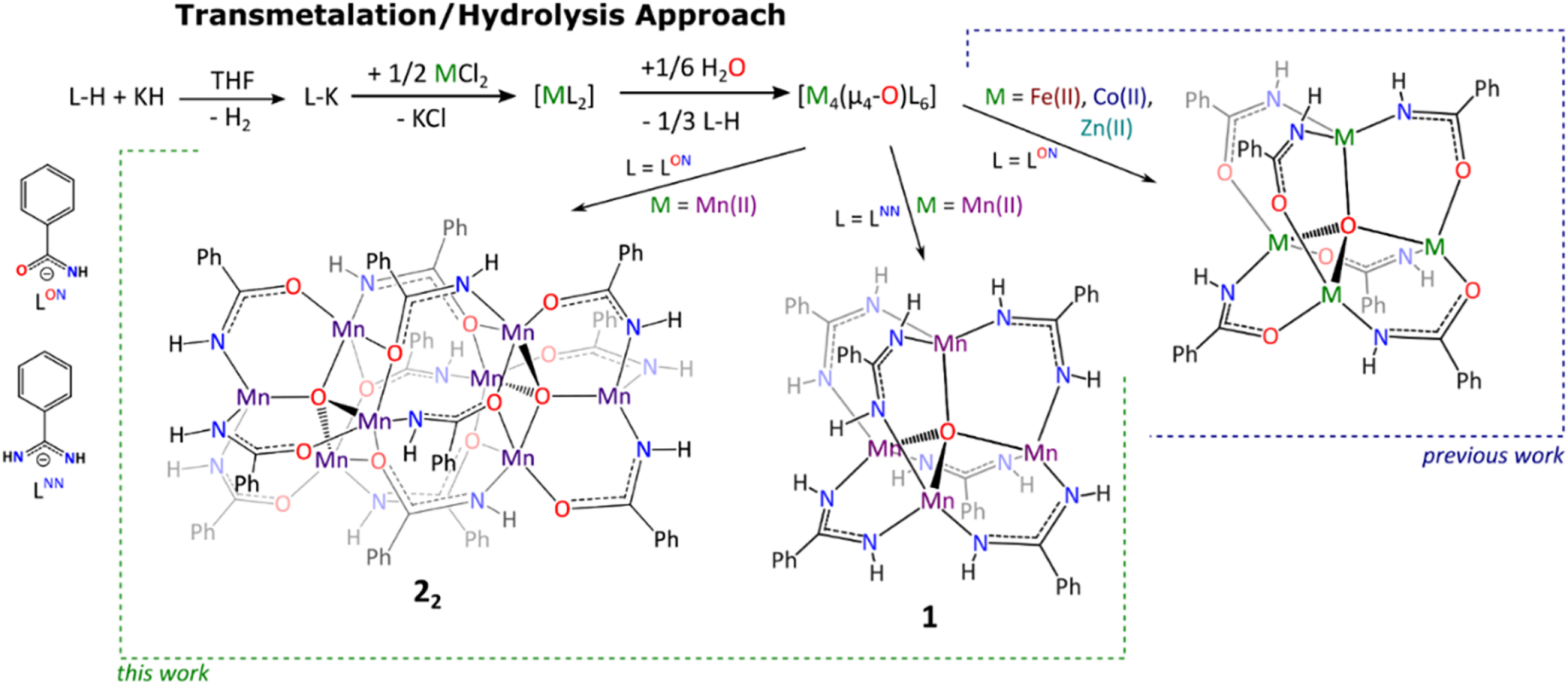
General scheme of the transmetalation/hydrolysis approach for the preparation of metal-oxido [M_4_(μ_4_-O)­(L)_6_]-type clusters.

### Structure and Self-Assembly of Mn­(II) Amidinato Cluster **1-Mn** and Its Zn­(II) Analogue

The molecular structure of cluster **1-Mn** is isostructural with the previously reported Zn­(II) analogue [Zn_4_(μ_4_-O)­(L^NN^)_6_].^36^ It comprises a highly symmetrical tetrahedral μ_4_-oxido-centered [Mn_4_^II^(μ_4_-O)]^6+^ core stabilized by six benzamidinate anions (Figure 3a) (for a detailed structure description, see the SI). Cluster **1-Mn** crystallizes from a DMF/THF solution (1:3, v/v) as a DMF-only solvate **1-Mn**·3DMF in the orthorhombic space group *Pna*2_1_. In the crystal lattice, there are two crystallographically independent molecules of **1-Mn** and six molecules of DMF. Both symmetry-unique clusters form similar **1-Mn**·3DMF solvates via N–H···O hydrogen bonds (the O···H distances are in the range of 2.309–2.540 Å), involving four of six DMF molecules (two DMF molecules form H-bonds with both crystallographically distinct clusters of **1-Mn**) (Figures 3b,c and S4a,b). Two additional DMF molecules are tightly enclosed within the crystal lattice (only one could be modeled from the SC-XRD data; see the SI), resulting in the overall crystal formula of {2­[Mn_4_(μ_4_-O)­(L^NN^)_6_]·4DMF}·2DMF. The individual solvated clusters **1-Mn**·3DMF further self-assemble into 1D zigzag chains via cooperative pairs of intermolecular N–H···N interactions (Figures 3g and S5a), similar to that previously observed in a series of amidato clusters [M_4_(μ_4_-O)­(L^ON^)_6_] (M = Fe, Zn, Co)^36,53^ (*vide infra*). These chains are further connected into a deformed honeycomb-like 2D supramolecular lattice (Figure 3e) via less specific intermolecular interactions involving phenyl rings in the distal secondary coordination sphere (Figure 3h).

**Figure 3:**
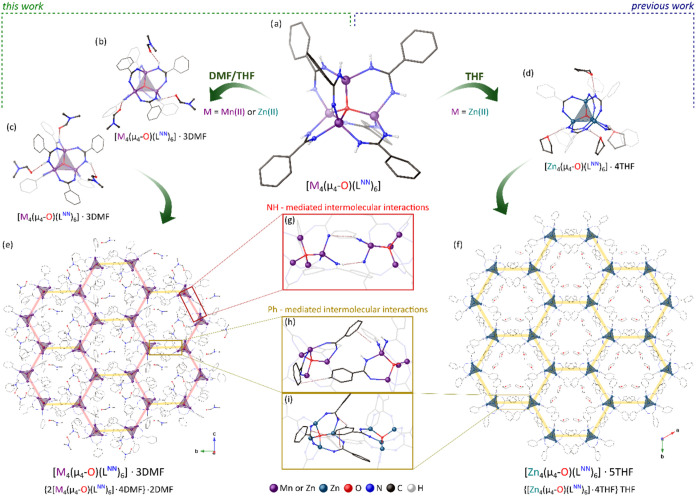
Structure and self-assembly of [M_4_(μ_4_-O)­(L^NN^)_6_]-type clusters (M = Zn­(II), Mn­(II)): (a) molecular structure; (b, c) H-bonded DMF solvates; (d) H-bonded THF solvate; (e) supramolecular 2D honeycomb-like layer formed by DMF-solvated metal-oxido clusters in [M_4_(μ_4_-O)­(L^NN^)_6_]·3DMF; (f) supramolecular 2D honeycomb layer formed by THF-solvated metal-oxido clusters in [Zn_4_(μ_4_-O)­(L^NN^)_6_]·5THF; (g–i) intermolecular noncovalent interactions between clusters within supramolecular 2D honeycomb layers.

Interestingly, the observed supramolecular structure of **1-Mn**·3DMF contrasts with the previous results obtained for Zn­(II) amidinato analogue [Zn_4_(μ_4_-O)­(L^NN^)_6_] (**1-Zn**),^36^ which crystallized from a THF solution as the [Zn_4_(μ_4_-O)­(L^NN^)_6_]·5THF solvate in the trigonal space group *P*31*c*. In this crystal structure, each zinc-oxido cluster forms hydrogen bonds with four THF molecules (Figure 3d), and the resulting **1-Zn**·4THF solvates self-assemble via cooperative CH···π and π–π noncovalent interactions mediated by the phenyl rings in the distal secondary coordination sphere (Figure 3i), affording a honeycomb-like supramolecular structure (Figure 3f). Another THF molecule is encapsulated within the crystal lattice without specific hydrogen interactions, leading to an overall **1-Zn**·5THF ({**1-Zn**·4THF}·THF) crystal formula.

To determine whether the observed formation of various solvatomorphs by Mn­(II) and Zn­(II) oxido analogues results from the differing chemical character of these metal centers, we carried out the synthesis and purification of cluster **1-Zn** according to the transmetalation/hydrolysis procedure utilizing ZnCl_2_ instead of MnCl_2_ (previously this cluster was obtained by an organometallic procedure and crystallized from THF solution).^36^ Eventually, crystallization of the product from a DMF/THF (1:3, v/v) solution led to the isolation of a new solvatomorph of **1-Zn**, in high yield, which was isostructural with **1-Mn**·3DMF. Crystals of **1-Zn**·3DMF were characterized by SC-XRD, FTIR, NMR spectroscopy, and elemental analysis (for details on the synthesis and characterization of **1-Zn**·3DMF, see the SI and Experimental Section).

The isolation of the isostructural DMF solvates for Zn­(II) and Mn­(II) amidinato [M_4_(μ_4_-O)­(L^NN^)_6_]-type clusters indicates that, in this case, their self-assembly is likely determined by the crystallization conditions rather than the type of metal center. Specifically, we found that the hydrogen bonding solvation by DMF or THF molecules competes with the formation of intercluster bridges via N–H···N interactions within the proximal secondary coordination sphere of the metal-oxide clusters, thereby affecting their interlocking. In the crystal structure of **1-Zn**·5THF, the H-bonded THF molecules efficiently block access to the interior of the clusters, hampering the formation of intermolecular N–H···N bridges (Figure 3d). Thus, THF-solvated clusters **1-Zn**·4THF are organized only by interactions involving phenyl rings in the distal secondary coordination sphere, forming 2D honeycomb layers with accurate trigonal symmetry (Figure 3f,i). In turn, DMF molecules in the solvatomorphs **1**·3DMF only partially block access to the interior of the metal clusters, which are organized by utilizing both the intermolecular N–H···N bridges (Figures 3g and S5) and the interactions involving phenyl rings (Figure 3h), leading to distorted 2D honeycomb-like layers with reduced symmetry to orthorhombic (Figure 3e). Furthermore, these observations contrast nicely with the structure of amidato clusters [M_4_(μ_4_-O)­(L^ON^)_6_] (M = Zn­(II), Co­(II), Fe­(II)), which in the same conditions (DMF/THF solution) self-assemble into 2D honeycomb layers with accurate trigonal symmetry utilizing only intermolecular N–H···X (X = N or O) bridges (*vide infra*, Figure 4h). Amidato ligands introduce O-donor centers to the secondary coordination sphere of metal-oxido clusters, which serve as stronger hydrogen-bond acceptors than the NH groups found exclusively in amidinato analogues. This likely increases the competitiveness of intermolecular N–H···X interactions over hydrogen-bonded solvation by DMF molecules in amidinato [M_4_(μ_4_-O)­(L^ON^)_6_]-type clusters.

**Figure 4:**
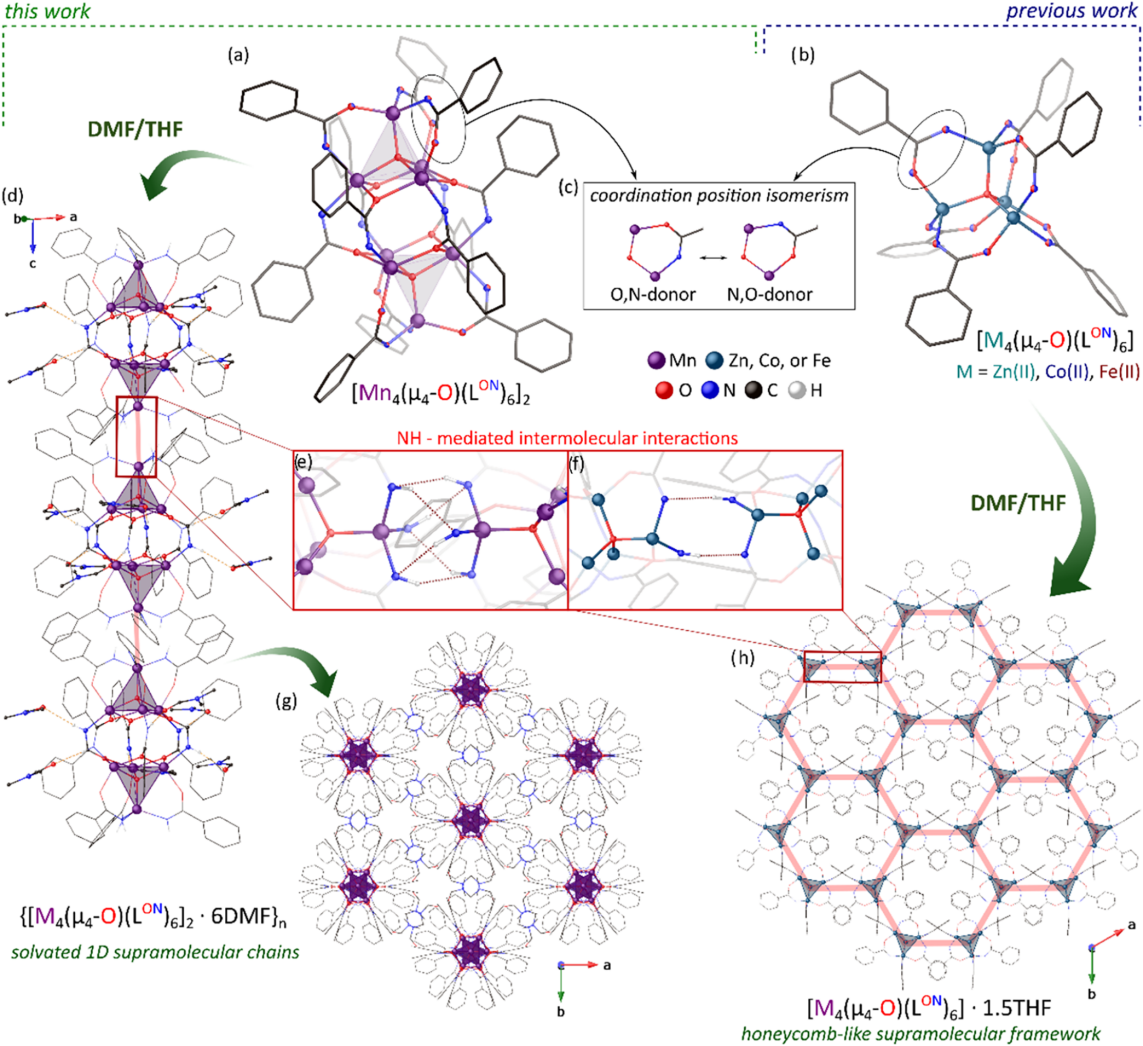
Structure and self-assembly of [Mn_4_(μ_4_-O)­(L^ON^)_6_]_2_ and [M_4_(μ_4_-O)­(L^ON^)_6_] (M = Zn­(II), Co­(II), Fe­(II)) clusters: (a) molecular structure of **2**_**2**_; (b) molecular structure of [M_4_(μ_4_-O)­(L^ON^)_6_] (M = Zn­(II), Co­(II), Fe­(II)); (c) coordination position isomerism of L^ON^ ligands in metal-oxido clusters; (d, g) self-assembly of **2**_**2**_ into the DMF-solvated 1D supramolecular chains via cooperative hydrogen bonds; (e, f) cooperative hydrogen-bond systems in supramolecular assemblies of [Mn_4_(μ_4_-O)­(L^ON^)_6_]_2_ and [M_4_(μ_4_-O)­(L^ON^)_6_] clusters [the presented case shows only NH groups; however, considering the possible position coordination isomerism of the ligands, the same sites may be also occupied by O atoms, which would reduce the number of hydrogen bonds]; (h) self-assembly of [M_4_(μ_4_-O)­(L^ON^)_6_] (M = Zn­(II), Co­(II), Fe­(II)) clusters into the THF-solvated honeycomb-like framework.

### Structure and Self-Assembly of Mn­(II) Amidato Cluster **2**_**2**_

In contrast to **1-Mn**, the molecular structure of Mn­(II)-oxido benzamidato analogue **2**_**2**_ is a centrosymmetric dimer composed of two [Mn_4_^II^(μ_4_-O)­(L^ON^)_6_] units connected via coordination bridges formed by organic ligands (Figure 4a). Both manganese-oxido [Mn_4_^II^(μ_4_-O)]^6+^ cores are arranged face-to-face with an approximately 60° axial rotation (e.g., the Mn01–Mn04–Mn04′-Mn03′ torsion angle is 62.90(3)°), resulting in a pseudo *S*_6_ molecular symmetry. The two [Mn_4_^II^(μ_4_-O)]^6+^ units in **2**_**2**_ are stabilized by an overall 12 monoanionic benzamidato ligands. Six of them adopt the μ_2_-κ^1^(*O*):κ^1^(*N*) coordination mode and are positioned along the external edges of both tetrahedral Mn_4_O units, similarly to what is observed in monomeric [M_4_(μ_4_-O)­(L)_6_]-type complexes. These ligands likely exhibit coordination position isomerism, acting either as *O*,*N*- or *N*,*O*-donors (Figure 4c), as previously demonstrated in the case of amidato [M_4_(μ_4_-O)­(L^ON^)_6_]-type clusters (M = Zn­(II), Co­(II), Fe­(II)).^53^ This phenomenon leads to positional disorder of the N and O atoms within the μ_2_-ligands in the crystal structure (for details, see the SI). The other six benzamidato ligands in complex **2**_**2**_ are located around the middle part of the cluster, adopting a μ_3_-μ_2_(*O*):κ^1^(*N*) coordination mode. Each of these ligands acts as a μ_3_-OCN bridge between both manganese-oxido units, forming a single Mn–N bond with one of the Mn_4_O tetrahedra and two Mn–O bonds with the second one (Figures 4a and 5b). Interestingly, the formation of μ_3_-OCN bridges between two manganese-oxido units in **2**_**2**_ dimer contrasts with most of the previously reported dimeric [M_4_(μ_4_-O)­(L)_6_]_2_-type structures of metal-oxido clusters, including the Mn­(II) cluster incorporating an *N*,*N*′-bidentate pyridin-azanido ligand^87^ (Figure 1b) and Co­(II)^88^ and Zn­(II)^61^ clusters stabilized by *O*,*O*′-donor carboxylato and carbamato ligands (Figure 5a). In all of these dimeric structures, the connection between two [M_4_(μ_4_-O)]^6+^ units is achieved through μ_2_-X-type bridges (X = O or N) mediated by single donor centers of ligands (Figure 5a). Notably, this connection is established via the simple formation of additional M–X bonds through one of the donor centers of the ligands. In contrast, the formation of μ_3_-OCN bridges requires a significant rearrangement of ligands compared with the initial monomeric [M_4_(μ_4_-O)­L_6_] species and, to the best of our knowledge, has previously been observed only in an exceptional pivalato Mg­(II) cluster [Mg_4_(μ_4_-O)­(O_2_CMe_3_)_6_]_2_.^89^ The type of bridging between metal-oxide units is also reflected in their spatial separation, which can be visualized by the (μ_4_-O)···(μ_4_-O) distances (Figure 5). In structures featuring μ_2_-X-type bridges, the respective distances are significantly shorter (3.45–3.64 Å) compared to the structures with μ_3_-OCN bridges (5.34–5.65 Å). The observed variation in the separation of [M_4_(μ_4_-O)]^6+^ units may have further implications for the physicochemical properties of the formed dimeric clusters.

**Figure 5:**
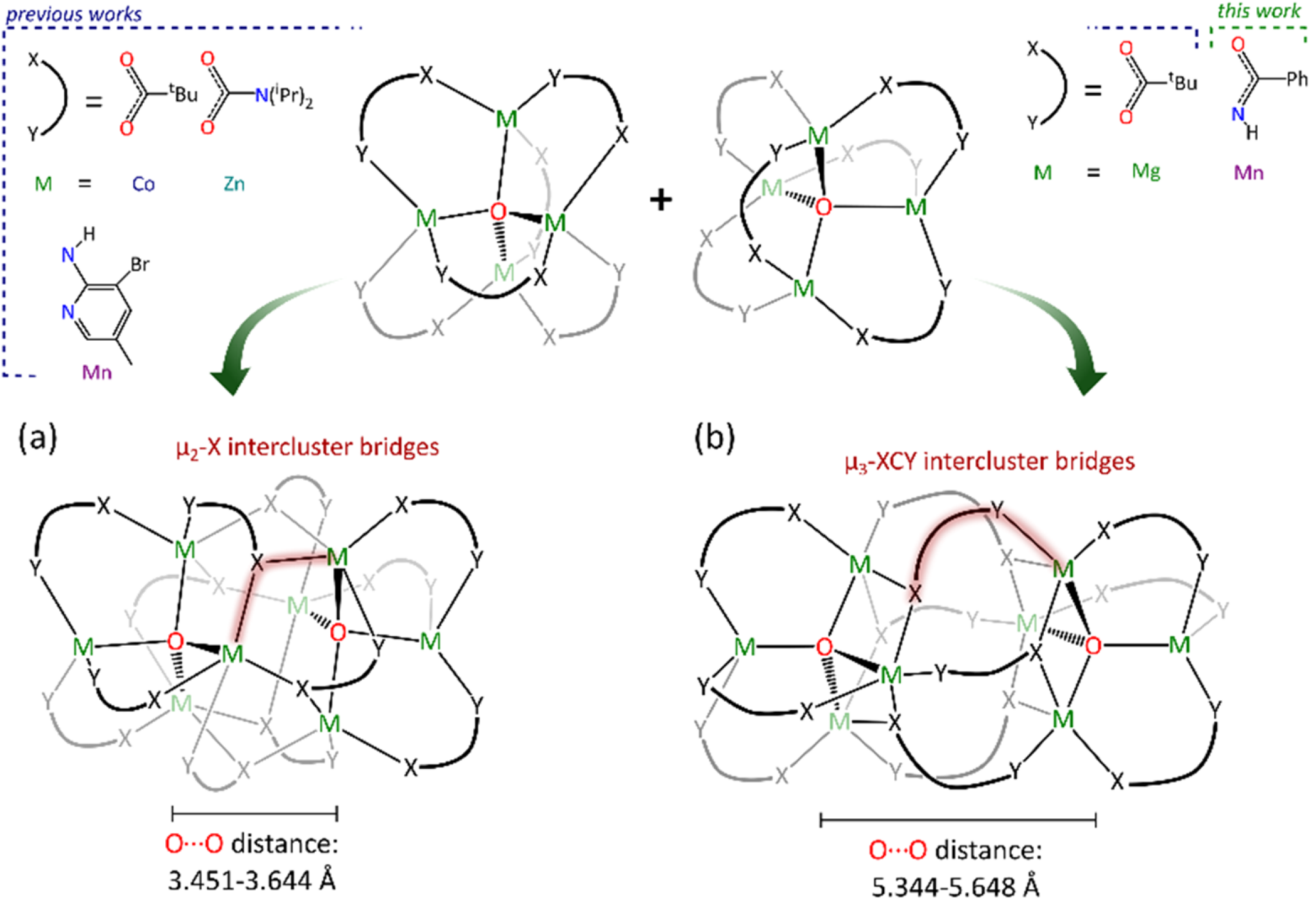
Two types of intercluster bridges: μ_2_-X (a) and μ_3_-XCY (b), formed within dimeric [M_4_(μ_4_-O)­(L)_6_]_2_-type clusters.

Complex **2**_**2**_ crystallizes as a DMF solvate **2**_**2**_·6DMF in the monoclinic *C*2/*c* space group. In the crystal lattice, molecules of **2**_**2**_ self-assemble into linear 1D supramolecular chains oriented along the crystallographic *c*-axis (Figure 4d). This organization is driven by intermolecular N–H···N­(O) hydrogen bonds, which act cooperatively, forming systems involving up to six interactions (Figures 4e and S5c). The NH groups function as both H-donor and H-acceptor sites, whereas the O atoms may only act as H-acceptors. Consequently, the number of hydrogen bonds may vary due to the coordination position isomerism of the μ_2_-L^ON^ ligands (*vide supra*). Additionally, each molecule of **2**_**2**_ is solvated by six DMF molecules via N–H···O hydrogen bonds with the bridging μ_3_-μ_2_(*O*):κ^1^(*N*) amidato ligands. The solvated 1D chains further form a close-packed lattice through additional intermolecular noncovalent interactions between aromatic rings (Figure 4g). The self-assembly of cluster **2**_**2**_ interestingly contrasts with the supramolecular structures formed by monomeric amidato [M_4_(μ_4_-O)­(L^ON^)_6_]-type analogues (M = Zn­(II), Co­(II), Fe­(II)), which organize into hexagonal honeycomb-like frameworks (Figure 4b,h). In these structures, the metal-oxido clusters form bridges involving only two N–H···N­(O) hydrogen bonds (compared with six between **2**_**2**_) within the proximal secondary coordination sphere, likely due to the presence of additional π···π interactions in the distal secondary coordination sphere (Figure 6). This leads to an angular arrangement of molecules instead of the linear architecture observed in the crystal structure of **2**_**2**_.

**Figure 6:**
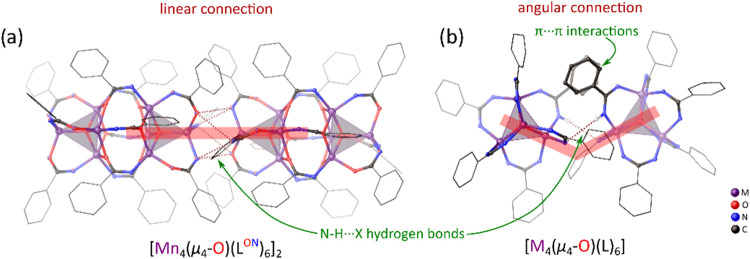
Comparison of intermolecular connections in supramolecular structures of dimeric (a) and monomeric (b) [M_4_(μ_4_-O)­(L^ON^)_6_]-type clusters.

## Conclusions

In conclusion, we have demonstrated the successful application of a previously developed transmetalation/hydrolysis procedure for the synthesis of new Mn­(II) oxido clusters, [Mn_4_(μ_4_-O)­(L^NN^)_6_] and [Mn_4_(μ_4_-O)­(L^ON^)_6_]_2_. Notably, while the synthesis of mixed-valence Mn­(II)/Mn­(III) oxido clusters has been readily achievable, access to homovalent Mn­(II) oxido clusters has remained challenging and was previously accomplished only serendipitously. The presented approach enabled not only the rational, on-demand synthesis of Mn­(II) oxido clusters but also provided control over their nuclearity, which was dictated by the electronic properties of the anchoring groups in the supporting ligands, affording monomeric cluster [Mn_4_(μ_4_-O)­(L^NN^)_6_] and dimeric cluster [Mn_4_(μ_4_-O)­(L^ON^)_6_]_2_ for *N*,*N*′-bidentate amidinato and *O*,*N*-bidentate amidato ligands, respectively. Furthermore, a detailed analysis of the supramolecular structures of these new complexes, in comparison with previously reported metal-oxido clusters, provided deeper insight into their noncovalent interaction-driven self-assembly. Notably, we found that [M_4_(μ_4_-O)­(L)_6_]_*x*_-type clusters (M = Zn­(II), Co­(II), Fe­(II), Mn­(II); x = 1 or 2) stabilized by both amidinato or amidato ligands exhibit a similar tendency for the self-assembly via specific cooperative intermolecular N–H···X (X = N or O) hydrogen bonds in their proximal secondary coordination sphere, which compete with H-bonded solvation by DMF or THF molecules. However, there are significant differences in the self-assembly of monomeric [M_4_(μ_4_-O)­(L)_6_] and dimeric [M_4_(μ_4_-O)­(L)_6_]_2_ clusters. In the former, the N–H···X intermolecular bridges are further reinforced by π···π interactions between aromatic rings in the distal secondary coordination sphere, leading to angular supramolecular connections and the formation of honeycomb-like frameworks. In contrast, the dimeric cluster [M_4_(μ_4_-O)­(L^ON^)_6_]_2_ lacks such π···π stabilization, likely due to the geometry of its secondary coordination sphere, and instead forms one-dimensional supramolecular chains through linear bridges involving six cooperative intermolecular N–H···X interactions. Overall, the presented studies not only introduce an efficient new approach to the synthesis of homovalent Mn­(II) oxido clusters but also reveal novel structural features within this family of compounds, which may have broader implications for the design and development of new functional materials.

## Experimental Section

### General Considerations

All manipulations were conducted under a dry, oxygen-free argon atmosphere using either standard Schlenk techniques or a glovebox. All reagents, benzamide (Sigma), benzamidine (Sigma), KH (Sigma), manganese­(II) chloride (ABCR), and zinc chloride (ABCR), were purchased from commercial vendors and used as received. Solvents were purified by using an MBraun SPS-5 system.

### Synthesis of **1-Mn**

Equimolar amounts of benzamidine (0.480 g, 4 mmol) and KH (0.160 g, 4 mmol) were placed in a Schlenk flask cooled to 0 °C and dispersed in THF (15 mL). After a few minutes, the reaction mixture was allowed to warm to room temperature and stirred for 24 h. Then, MnCl_2_ (0.250 g, 2 mmol) was added. After another 8 h of stirring, 9 μL (0.5 mmol) of Millipore water was added, followed by 5 mL of DMF. The reaction mixture was stirred for an additional 24 h. The product was isolated as yellow crystals after filtration and crystallization in the presence of hexane vapor at room temperature (Yield = 73%, 348 mg). ^1^H NMR (600 MHz, *d*_8_-THF): δ [ppm] = 11–9 (bs, Ph), 6–3 (bs, NH); FTIR (ATR): ν/cm^–1^ = 3356 (w), 3053 (w), 1587 (m), 1539 (s), 1506 (m), 1466 (m), 1407 (w), 1339 (w), 1320 (w), 1259 (m), 1119 (w), 1078 (w), 1029 (w), 924 (w), 788 (w), 745 (m), 658 (m), 631 (w), 582 (w), 465 (s). Elemental analysis: calcd (%) for **1-Mn**·2.73DMF (C_50.2_H_61.1_N_14.7_O_3.7_Mn_4_) (1150.18): C 52.41, H 5.36, N 17.94;found (%): C 52.40, H 5.51, N 17.43.

### Synthesis of **1-Zn**

The same procedure as for **1-Mn** was applied, but with ZnCl_2_ (0.272 g, 2 mmol) instead of MnCl_2_. The product was isolated as colorless crystals after filtration and crystallization in the presence of hexane vapor at room temperature (Yield = 77%, 381 mg). ^1^H NMR (600 MHz, *d*_8_-THF): δ [ppm] = 7.62–7.55 (m, 12H, Ph), 7.34–7.25 (m, 18H, Ph), 4.71 (s, 12H, NH); ^13^C NMR (150 MHz, CDCl_3_): δ [ppm] = 174.54 (NCN), 144.78 (^IV^C_Ar_), 129.37 (C_Ar_), 128.91 (C_Ar_), 126.74 (C_Ar_); FTIR (ATR): ν/cm^–1^ = 3363 (w), 3063 (w), 1599 (s), 1557 (s), 1502 (m), 1450 (s), 1342 (w), 1320 (w), 1303 (w), 1278 (w), 1233 (m), 1185 (w), 1158 (w), 1131 (m), 1069 (w), 1028 (w), 1002 (w), 934 (w), 829 (w), 804 (w), 779 (w), 695 (s), 672 (s), 620 (w), 604 (w), 521 (m), 487 (s), 440 (w), 408 (w). Elemental analysis: calcd (%) for **1-Zn**·2.64DMF (C_49.9_H_60.5_N_14.6_O_3.6_Zn_4_) (1185.37): C 50.58, H 5.14, N 17.30; found (%): C 50.63, H 5.18, N 17.25.

### Synthesis of **2**_**2**_

The same procedure as that for **1-Mn** was applied, but with benzamide (0.484 g, 4 mmol) instead of benzamidine. The obtained suspension was separated, and the precipitate was extracted twice with 10 mL of THF/DMF mixture (1:3, v/v). All three solutions were combined and crystallized in the presence of hexane vapor at room temperature, yielding yellow crystals of the product (Yield = 69%, 332 mg). FTIR (ATR): ν/cm^–1^ = 3340 (w), 3053 (w), 1610 (w), 1590 (m), 1540 (s), 1436 (m), 1410 (m), 1250 (w), 1235 (w), 1200 (m), 1156 (w), 1118 (m), 1073 (w), 1058 (w), 1026 (w), 929 (w), 851 (w), 809 (w), 699 (s), 618 (w), 574 (w), 531 (w), 461 (m), 434 (w). Elemental analysis: calcd (%) for **2**_**2**_·6.49DMF (C_103.5_H_117.4_N_18.5_O_20.5_Mn_8_) (2387.46): C 52.05, H 4.96, N 10.85; found (%): C 51.85, H 5.01, N 10.75.

### Characterization

FTIR spectra were measured with a Bruker Tensor II spectrometer by using the ATR technique. Elemental analyses were performed using a UNICUBE elemental analyzer (Elementar Analysensysteme GmbH). The ^1^H and ^13^C NMR spectra were acquired on a JEOL JNM-ECZL (600 MHz) spectrometer. The Continuous Shape Measurement (CShM) parameters were calculated utilizing SHAPE software.^90^ The closer the calculated CShM is to zero, the closer the given coordination environment is to the ideal given geometry.^91^

### X-ray Structure Determination

The crystals were selected under Paratone-N oil, mounted on the nylon loops, and positioned in the cold stream on the diffractometer. The X-ray data for complexes **1-Mn, 1-Zn**, and **2**_**2**_ were collected at 100(2)K on a SuperNova Agilent diffractometer using graphite monochromated MoKa radiation (λ = 0.71073 Å). The data were processed with CrysAlisPro.^92^ The structures were solved by direct methods using the SHELXS-97 program and were refined by full-matrix least-squares on *F* ^2^ using the program SHELXL-2018/3 ^93^ implemented in the Olex2 ^94^ suite. All non-hydrogen atoms were refined with anisotropic displacement parameters. Hydrogen atoms were added to the structure model at geometrically idealized coordinates and refined as riding atoms.

## Supplementary Material









## Notes

Crystallographic data and crystal structure analysis, FTIR and NMR spectra (PDF)
**1-Mn** (CIF)
**1-Zn** (CIF)
**2_2_** (CIF)
