# Supplementary material for: Discrete μ4‑Oxido Mn(II) Clusters with Controlled Nuclearity: On-Demand Synthesis, Molecular and Self-Assembled Structures
Source: ACS Omega. 2025 Sep 26;10(39):45718–28. doi: 10.1021/acsomega.5c06133 (PMC12508945; doi:10.1021/acsomega.5c06133)
Supplement: Supplementary file 1 [file ao5c06133_si_001.pdf]

Supporting Information

Discrete μ_4-Oxido Mn(II) Clusters with Controlled Nuclearity: On-Demand Synthesis, Molecular and Self-Assembled Structures

Dominik Jabłoński[†], Maciej Jacyna[†], Michał Terlecki[†], Arkadiusz Kornowicz[‡], Iwona Justyniak[‡], and Janusz Lewiński[†‡]**

[†]Faculty of Chemistry, Warsaw University of Technology, Noakowsiego 3, 00-664 Warsaw, Poland
[‡]Institute of Physical Chemistry, Polish Academy of Sciences, Kasprzaka 44/52, 01-224 Warsaw, Poland

*michal.terlecki@pw.edu.pl
*janusz.lewinski@pw.edu.pl

Table of contents

1. Crystallographic data and crystal structure analysis S2

2. FTIR Spectra S15

3. NMR Spectra S16

4. PXRD analysis S17

1. Crystallographic data and crystal structure analysis

Description of the molecular structure of 1-Mn. The molecular structure of cluster **1-Mn** is isostructural with previously reported Zn(II) analog [Zn_4(μ_4-O)(L^{NN})$_6$].[1] It comprises a highly symmetrical tetrahedral μ_4-oxido-centered [Mn^{II}_4(μ_4-O)]$^{6+}$ core (the Continuous Shape Measurement (CShM)[2] parameters for Mn_4O tetrahedra units CShM(T_d) are 0.076 and 0.038 for O1 and O2 centers, respectively; see Table S4) stabilized by six benzamidinate anions (Figure 3a). The Mn-O bond lengths are in the range of 2.017(5)-2.033(4) Å, which is similar to that of previously reported for amidinato [Mn^{II}_4(μ_4-O)L_6]-type complexes[3–5] (2.014(2)-2.028(2) Å). The monoanionic L^{NN} ligands adopt a μ_2-κ^1(N):κ^1(N′) coordination mode and are uniformly distributed along each edge of the Mn_4O tetrahedron. The Mn-N bond lengths (2.073(6)-2.118(6) Å) fall within the typical range observed in similar Mn(II)-oxido amidinato clusters[3–5] (2.104-2.124 Å). All Mn(II) centers adopt similar tetrahedral ON_3 coordination environments (the CShM(T_d) parameters are in the range of 0.035-0.136, see Table S8 and Figure S4).

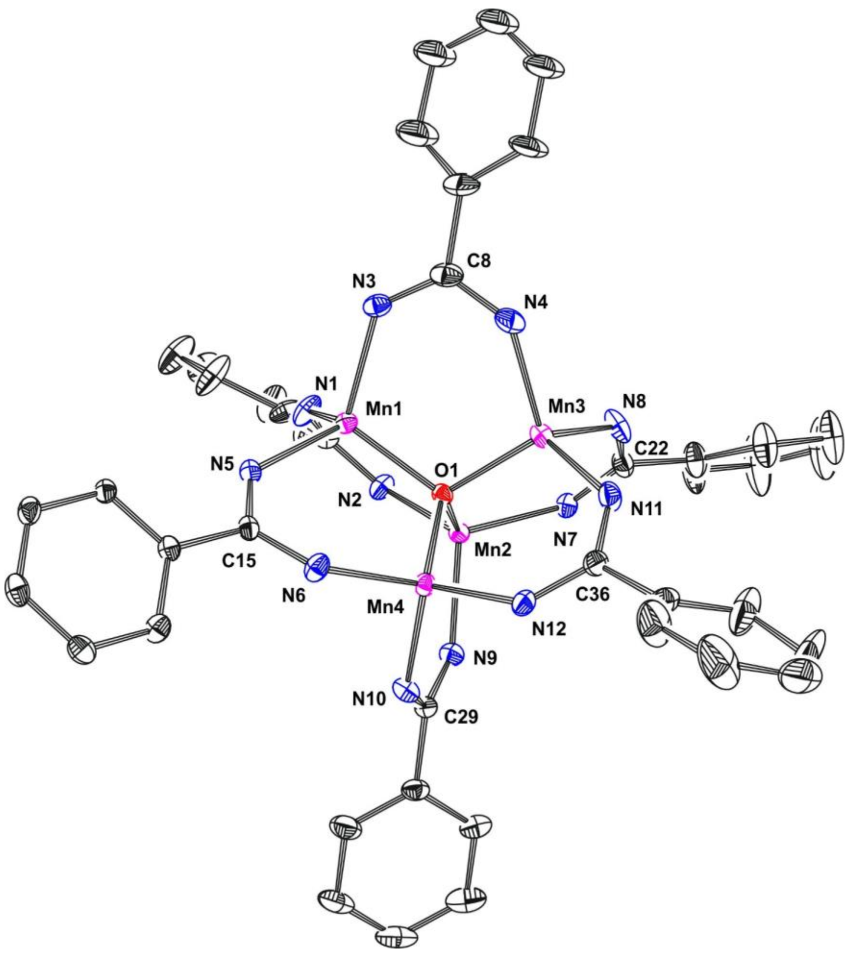

Figure S1. The molecular structure of **1-Mn** with thermal ellipsoids set at 30% probability. Hydrogen atoms have been omitted for clarity.

Table S1. Crystallographic data and Structure refinement parameters for **1-Mn**.

Empirical formula	$C_{97.78}H_{118.51}Mn_8N_{29}O_7$	
Formula weight	2251.60	
Temperature	100(2) K	
Wavelength	0.71073 Å	
Crystal system	Orthorhombic	
Space group	*P*n2$_1$a	
Unit cell dimensions	*a* = 25.4787(4) Å	*α* = 90°
	b = 28.5846(4) Å	*β* = 90°
	c = 15.2281(2) Å	*γ* = 90°
Volume	11090.6(3) Å ^{3}	
Z	4	
Density (calculated)	1.348 Mg/m^3	
Absorption coefficient	0.943 mm^{-1}	
F(000)	4657	
Crystal size	0.17 x 0.13 x 0.08 mm^3	
Theta range for data collection	2.111 to 30.187°.	
Index ranges	-35<=*h*<=35, -39<=*k*<=38, -21<=*l*<=19	
Reflections collected	107128	
Independent reflections	28509 [*R(int)* = 0.0989]	
Completeness to theta = 25.242°	99.9 %	
Refinement method	Full-matrix least-squares on F^2	
Data / restraints / parameters	28509 / 410 / 1435	
Goodness-of-fit on F^2	1.062	
Final R indices [I>2sigma(I)]	R_1 = 0.0656, wR_2 = 0.1648	
R indices (all data)	R_1 = 0.0905, wR_2 = 0.1821	
Absolute structure parameter	0.38(2)	
Largest diff. peak and hole	0.802 and -0.561 e.Å^{-3}	

Table S2. Selected bond lengths (Å) and angles (deg) for **1-Mn**.

Bond Lengths (Å)

Mn1-O1	2.025(4)	Mn3-N8	2.084(6)	Mn5-N17	2.107(6)
Mn2-O1	2.026(4)	Mn3-N11	2.103(6)	Mn6-N14	2.102(6)
Mn3-O1	2.021(4)	Mn4-N6	2.080(5)	Mn6-N19	2.101(6)
Mn4-O1	2.022(4)	Mn4-N10	2.118(6)	Mn6-N21	2.098(6)
Mn1-N1	2.098(6)	Mn4-N12	2.095(6)	Mn7-N16	2.082(6)
Mn1-N3	2.109(6)	Mn5-O2	2.017(5)	Mn7-N20	2.095(6)
Mn1-N5	2.103(5)	Mn6-O2	2.033(4)	Mn7-N23	2.089(6)
Mn2-N2	2.085(6)	Mn7-O2	2.030(4)	Mn8-N18	2.111(5)
Mn2-N7	2.090(5)	Mn8-O2	2.029(5)	Mn8-N22	2.093(6)
Mn2-N9	2.112(6)	Mn5-N13	2.101(7)	Mn8-N24	2.102(5)
Mn3-N4	2.073(6)	Mn5-N15	2.101(7)		

Bond Angles (deg)

Mn1-O1-Mn2	109.6(2)	N1-Mn1-N3	104.0(3)	N13-Mn5-	107.7(3)
Mn1-O1-Mn3	111.4(2)	N1-Mn1-N5	107.7(2)	N13-Mn5-N17	106.8(3)
Mn1-O1-Mn4	107.8(2)	N3-Mn1-N5	113.5(2)	N15-Mn5-N17	109.4(2)
Mn2-O1-Mn3	108.4(2)	N2-Mn2-N7	105.6(2)	N14-Mn6-N19	105.0(2)
Mn2-O1-Mn4	113.3(2)	N2-Mn2-N9	110.6(2)	N14-Mn6-N21	113.2(2)
Mn3-O1-Mn4	106.4(2)	N7-Mn2-N9	106.7(2)	N19-Mn6-N21	104.7(2)
Mn5-O2-Mn6	109.5(2)	N4-Mn3-N8	109.5(3)	N16-Mn7-N20	112.2(3)
Mn5-O2-Mn7	108.5(2)	N4-Mn3-N11	110.3(3)	N16-Mn7-N23	106.2(3)
Mn5-O2-Mn8	111.7(2)	N8-Mn3-N11	104.7(3)	N20-Mn7-N23	107.9(3)
Mn6-O2-Mn7	110.0(2)	N6-Mn4-N10	105.2(2)	N18-Mn8-N22	109.4(2)
Mn6-O2-Mn8	110.8(2)	N6-Mn4-N12	109.6(2)	N18-Mn8-N24	108.1(2)
Mn7-O2-Mn8	106.3(2)	N10-Mn4-N12	109.1(2)	N22-Mn8-N24	105.0(2)

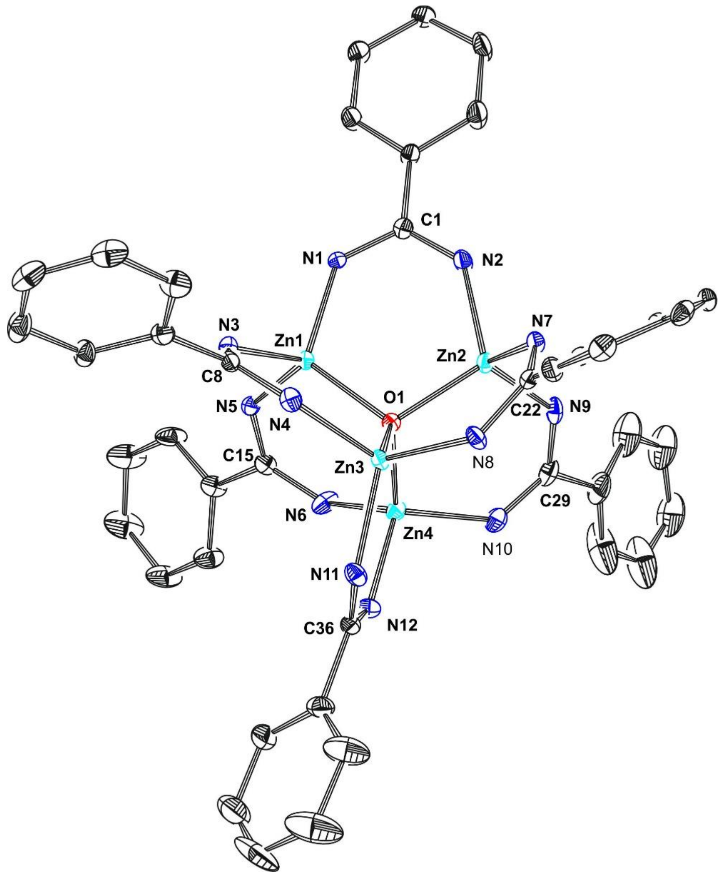

Figure S2. The molecular structure of **1-Zn** with thermal ellipsoids set at 30% probability. Hydrogen atoms have been omitted for clarity.

Table S3. Crystallographic data and Structure refinement parameters for **1-Zn**.

Empirical formula	$C_{99.59}H_{115.86}Zn_8N_{29}O_7$	
Formula weight	2350.46	
Temperature	100(2) K	
Wavelength	0.71073 Å	
Crystal system	Orthorhombic	
Space group	$Pna2_1$	
Unit cell dimensions	a = 25.4574(3) Å	α = 90°
	b = 15.08700(10) Å	β = 90°
	c = 28.3755(3) Å	γ = 90°
Volume	10898.34(19) $Å^3$	
Z	4	
Density (calculated)	1.433 Mg/m^3	
Absorption coefficient	1.794 mm^{-1}	
F(000)	4849	
Crystal size	0.22 x 0.16 x 0.10 mm^3	
Theta range for data collection	2.659 to 29.978°.	
Index ranges	-32<=h<=32, -19<=k<=19, -36<=l<=36	
Reflections collected	86856	
Independent reflections	23066 [$R(int)$ = 0.0360]	
Completeness to theta = 25.242°	99.8 %	
Refinement method	Full-matrix least-squares on F^2	
Data / restraints / parameters	23066 / 347 / 1429	
Goodness-of-fit on F^2	1.020	
Final R indices [I>2sigma(I)]	R_1 = 0.0395, wR_2 = 0.0930	
R indices (all data)	R_1 = 0.0497, wR_2 = 0.0987	
Absolute structure parameter	0.008(3)	
Largest diff. peak and hole	0.719 and -0.802 e.$Å^{-3}$	

Table S4. Selected bond lengths (Å) and angles (deg) for **1-Zn**.

**Bond Lengths (Å)**					
Zn1-O1	1.965(3)	Zn3-N8	1.980(5)	Zn5-N17	2.006(5)
Zn2-O1	1.960(4)	Zn3-N11	2.000(5)	Zn6-N16	1.992(5)
Zn3-O1	1.949(4)	Zn4-N6	2.002(5)	Zn6-N19	1.991(5)
Zn4-O1	1.950(3)	Zn4-N10	1.977(5)	Zn6-N21	1.992(5)
Zn1-N1	1.982(5)	Zn4-N12	1.993(5)	Zn7-N18	2.000(5)
Zn1-N3	2.016(5)	Zn5-O2	1.964(4)	Zn7-N20	1.997(5)
Zn1-N5	1.986(4)	Zn6-O2	1.957(3)	Zn7-N23	2.003(5)
Zn2-N2	1.998(5)	Zn7-O2	1.968(4)	Zn8-N14	2.010(5)
Zn2-N7	2.006(5)	Zn8-O2	1.951(4)	Zn8-N22	2.005(5)
Zn2-N9	2.020(5)	Zn5-N13	2.004(5)	Zn8-N24	1.996(5)
Zn3-N4	2.019(5)	Zn5-N15	2.012(5)		
**Bond Angles (deg)**					
Zn1-O1-Zn2	109.2 (2)	N1-Zn1-N3	109.6(2)	N13-Zn5-N15	104.1(2)
Zn1-O1-Zn3	112.9(2)	N1-Zn1-N5	105.8(2)	N13-Zn5-N17	112.1(2)
Zn1-O1-Zn4	108.2(2)	N3-Zn1-N5	105.4(2)	N15-Zn5-N17	104.3(2)
Zn2-O1-Zn3	108.2(2)	N2-Zn2-N7	105.7(2)	N16-Zn6-N19	105.8(2)
Zn2-O1-Zn4	111.2(2)	N2-Zn2-N9	104.4(2)	N16-Zn6-N21	110.3(2)
Zn3-O1-Zn4	107.1(2)	N7-Zn2-N9	113.1(2)	N19-Zn6-N21	105.3(2)
Zn5-O2-Zn6	109.6(2)	N4-Zn3-N8	105.4(2)	N18-Zn7-N20	103.5(2)
Zn5-O2-Zn7	110.8(2)	N4-Zn3-N11	108.0(2)	N18-Zn7-N23	108.6(2)
Zn5-O2-Zn8	110.1(2)	N8-Zn3-N11	108.6(2)	N20-Zn7-N23	108.0(2)
Zn6-O2-Zn7	106.6(2)	N6-Zn4-N10	107.6(2)	N14-Zn8-N22	107.5(2)
Zn6-O2-Zn8	108.4(2)	N6-Zn4-N12	104.0(2)	N14-Zn8-N24	105.8(2)
Zn7-O2-Zn8	111.3(2)	N10-Zn4-N12	108.8(2)	N24-Zn8-Zn24	107.7(2)

Description of the molecular structure of 2_2. The molecular structure of 2_2 is a centrosymmetric dimer, composed of two $[Mn^{II}_4(\mu_4\text{-}O)(L^{ON})_6]$ units connected *via* coordination bridges formed by organic ligands (Figure 4a). Both manganese-oxido $[Mn^{II}_4(\mu_4\text{-}O)]^{6+}$ cores are arranged face-to-face with an approximately 60° axial rotation (e.g., the Mn01-Mn04-Mn04'-Mn03' torsion angle is 62.90(3)°), resulting in a pseudo S_6 molecular symmetry. Six Mn(II) centers in the middle of the cluster adopt a distorted trigonal bipyramidal coordination geometry (CShM(C_{3v}) values range are in the range of 1.548-1.668; see Table S9 and Figure S4d), while the two distal Mn(II) centers exhibit a tetrahedral coordination environment (SChM(T_d) is 0.087; see Table S8 and Figure S4c). The Mn-O bonds between central μ_4-O^{2-} anions and trigonal bipyramidal Mn(II) centers are marginally longer (2.051(5) - 2.061(5) Å) than the Mn–O bonds with the tetrahedral Mn(II) centers (1.956(5) Å). The typical Mn-(μ_4-O) bond lengths in known amidinato $[Mn^{II}_4(\mu_4\text{-}O)L_6]$-type complexes (2.014(2)–2.028(2) Å),[3–5] as well as in complex **1-Mn** reported herein (2.015-2.039 Å) (*vide supra*), fall between the longer and shorter Mn-(μ_4-O) bond lengths in 2_2.

The two $[Mn^{II}_4(\mu_4\text{-}O)]^{6+}$ units in 2_2 are stabilized by overall twelve monoanionic benzamidato ligands. Six of them adopt the μ_2-$\kappa^1(O)$:$\kappa^1(N)$ coordination mode and are positioned along the external edges of both tetrahedral Mn_4O units, similarly to what is observed in monomeric $[M_4(\mu_4\text{-}O)(L)_6]$-type complexes. These ligands likely exhibit coordination position isomerism, acting either as *O,N*- or *N,O*-donors (Figure 4c), as previously demonstrated in the case of amidato $[M_4(\mu_4\text{-}O)(L^{ON})_6]$-type clusters (M = Zn(II), Co(II), Fe(II)).[6] This phenomenon leads to positional disorder of the N and O atoms within the μ_2-ligands in the crystal structure of 2_2. Such a view is supported by the modeled partial N/O occupancies of these positions during crystal structure refinement, as well as by the analysis of bond distances. The positions N6/O6a, N8a/O8, N3a/O3, N5/O5a, N7/O7a, and N6a/O6 in the crystal structure of compound 2_2 were refined by constraining the coordinates of both O and N atoms within each position and setting their partial occupancies to sum to 1. Additionally, within each individual μ_2-ligand, the N and O atoms were assigned in a way consistent with the presence of amide groups (i.e., each ligand contains one nitrogen and one oxygen atom in total). This approach leads to better refinement of the structure. The best refinement results were obtained with estimated occupancies of $N_{0.29}O_{0.71}$, $N_{0.71}O_{0.29}$, and $N_{0.62}O_{0.38}$ for positions N6/O6A, N5/O5A, and N7/O7A, respectively, and corresponding $N_{0.71}O_{0.29}$, $N_{0.29}O_{0.71}$, and $N_{0.38}O_{0.62}$ for N6/O6A, N5/O5A, and O8/N8A in the second donor centers of respective ligands (see, Table S6). The Mn-X bonds (X = O or N) formed by these ligands fall in two marginally different ranges of 2.091(6)-2.099(6) Å and 2.101(6)-2.126(5) Å, which correlates with chemical occupancy of the donor atoms, i.e., the longer bonds are formed with the donor centers characterized by a higher N:O ratio, (see, Table S6). The length of the longer Mn-X bonds match well with typical Mn−N bond lengths in known amidinato $[Mn^{II}_4(\mu_4\text{-}O)L_6]$-type complexes (2.104(2)-2.124(2) Å).[3–5] However, a proper comparison of Mn-X bond lengths in 2_2 with typical Mn−O bond lengths is hindered by the scarcity of Mn(II) complexes featuring metal centers with tetrahedral coordination environment featuring anionic O-donor ligands. Note that the metal-ligand bond length may be significantly affected by the coordination sphere geometry. The other six benzamidato ligands in complex 2_2 are located around the middle part of the cluster acting as a μ_3-OCN bridge between both manganese-oxido units by forming a single Mn-N bond with one of the Mn_4O tetrahedra and two Mn-O bonds with the second one (Figure 4a and 5b). Both Mn-

O (2.180(5)-2.205(5) Å) and Mn-N (2.133(6)-2.139(6) Å) bonds are slightly longer than those in μ_2-benzamidato ligands (*vide supra*).

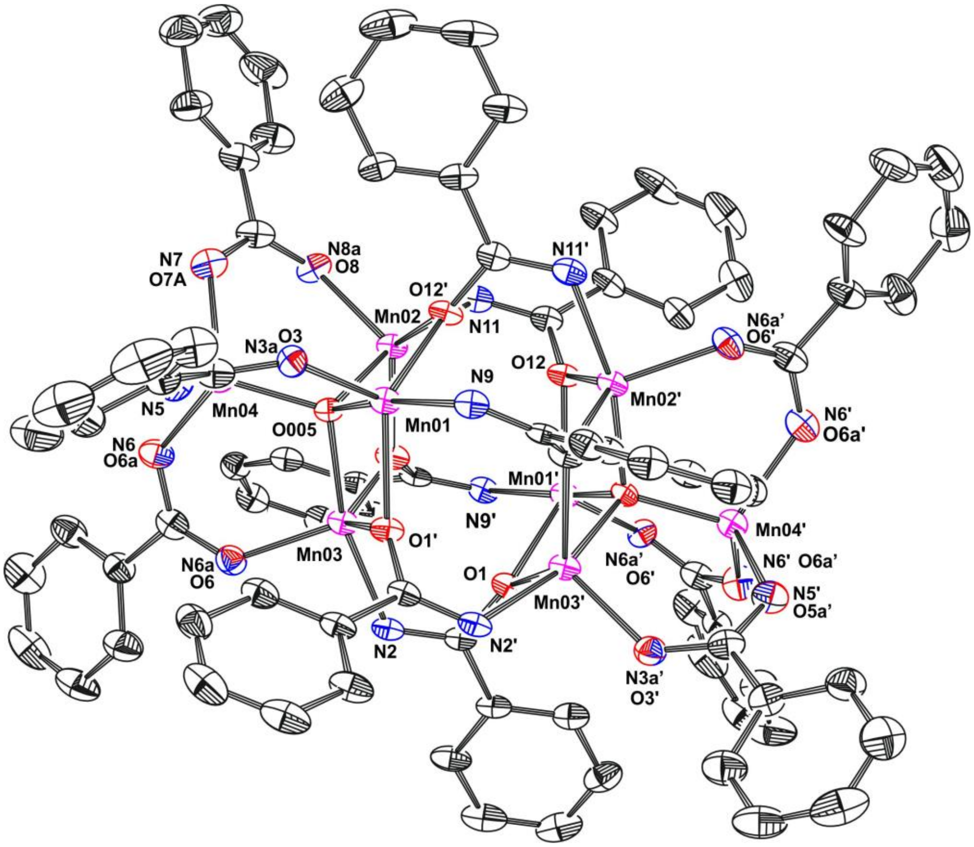

Figure S3. The molecular structure of $\mathbf{2_2}$ with thermal ellipsoids set at 30% probability. Hydrogen atoms have been omitted for clarity. Operators for generated equivalent atoms: (*-x+1,y,-z+1/2*).

Table S5. Crystallographic data and Structure refinement parameters for **2₂**.

Empirical formula	$C_{102}H_{108}Mn_8N_{18}O_{20}$	
Formula weight	2345.58	
Temperature	100(2) K	
Wavelength	0.71073 Å	
Crystal system	Monoclinic	
Space group	*C*2/c	
Unit cell dimensions	*a* = 26.4314(14) Å	*α* = 90°.
	b = 15.3658(9) Å	*β* = 93.709(5)°.
	c = 26.3947(18) Å	*γ* = 90°.
Volume	10697.5(11) $Å^3$	
Z	4	
Density (calculated)	1.456 Mg/m^3	
Absorption coefficient	0.987 mm^{-1}	
F(000)	4824	
Theta range for data collection	2.114 to 26.500°.	
Index ranges	-33<=*h*<=33, -19<=*k*<=19, -33<=*l*<=33	
Reflections collected	11086	
Independent reflections	11086 [*R(int)* = 0.0756]	
Completeness to theta = 25.242°	99.9 %	
Refinement method	Full-matrix least-squares on F^2	
Data / restraints / parameters	11086 / 0 / 677	
Goodness-of-fit on F^2	0.985	
Final R indices [I>2sigma(I)]	R_1 = 0.0924, wR_2 = 0.2409	
R indices (all data)	R_1 = 0.1734, wR_2 = 0.3088	
Largest diff. peak and hole	2.522 and -0.831 e. $Å^{-3}$	

Table S6. Chemical occupancy and Mn-X bond lengths for donor atoms of μ_2-ligands in the crystal structure of **2₂**.

Position	Occupancy	Mn-X bond length [Å]
N6/O6a	$O_{0.71}N_{0.29}$	2.091(6)
N8a/O8	$O_{0.62}N_{0.38}$	2.096(6)
N3a/O3	$O_{0.71}N_{0.29}$	2.099(6)
N5/O5a	$N_{0.71}O_{0.29}$	2.101(6)
N7/O7a	$N_{0.62}O_{0.38}$	2.115(6)
N6a/O6	$N_{0.71}O_{0.29}$	2.126(5)

Table S7. Analysis of the coordination sphere geometry of O^{2-} anions within [(μ_4-O)M_4] units in the structures **1-Mn**, **1-Zn**, and **2_2** using the Continuous Shape Measurement (CShM) with tetrahedron (T_d), vacant trigonal bipyramid (C_{3v}), and square (D_{4h}) symmetry.

Structure	Central O atom	CShM(T_d)	CShM(C_{3v})	CShM(D_{4h})
**1-Mn**	O1	0.076	3.356	31.001
	O2	0.038	3.172	32.201
**1-Zn**	O1	0.062	3.351	31.212
	O2	0.032	3.099	32.552
**2_2**	O005	0.080	3.284	33.294

Table S8. Analysis of the coordination sphere geometry of 4-coordinated metal centers in the structures **1-Mn**, **1-Zn**, and **2_2** using the Continuous Shape Measurement (CShM) with tetrahedron (T_d), vacant trigonal bipyramid (C_{3v}), and square (D_{4h}) symmetry.

Structure	Central M atom	CShM(T_d)	CShM(C_{3v})	CShM(D_{4h})
**1-Mn**	Mn1	0.117	2.760	31.897
	Mn2	0.096	3.333	31.421
	Mn3	0.083	3.233	31.868
	Mn4	0.059	3.080	32.798
	Mn5	0.035	3.351	32.503
	Mn6	0.136	2.948	30.692
	Mn7	0.071	3.254	31.655
	Mn8	0.107	3.072	30.994
**1-Zn**	Zn1	0.105	3.209	31.566
	Zn2	0.124	2.731	31.303
	Zn3	0.058	3.040	32.909
	Zn4	0.096	3.175	32.335
	Zn5	0.147	2.862	30.919
	Zn6	0.081	3.061	31.823
	Zn7	0.134	2.992	31.277
	Zn8	0.050	3.152	33.186
**2_2**	Mn04	0.087	3.029	33.131

Table S9. Analysis of the coordination sphere geometry of 5-coordinated metal centers in structure **2₂** using the Continuous Shape Measurement (CShM) with pentagon (D_{5h}), trigonal bipyramid (D_{3h}), and square pyramid (C_{4v}) symmetry.

Structure	Central M atom	CShM(D_{5h})	CShM(D_{3v})	CShM(D_{4h})
**2_2**	Mn01	30.625	1.548	3.143
	Mn02	30.532	1.623	2.985
	Mn03	30.215	1.668	2.965

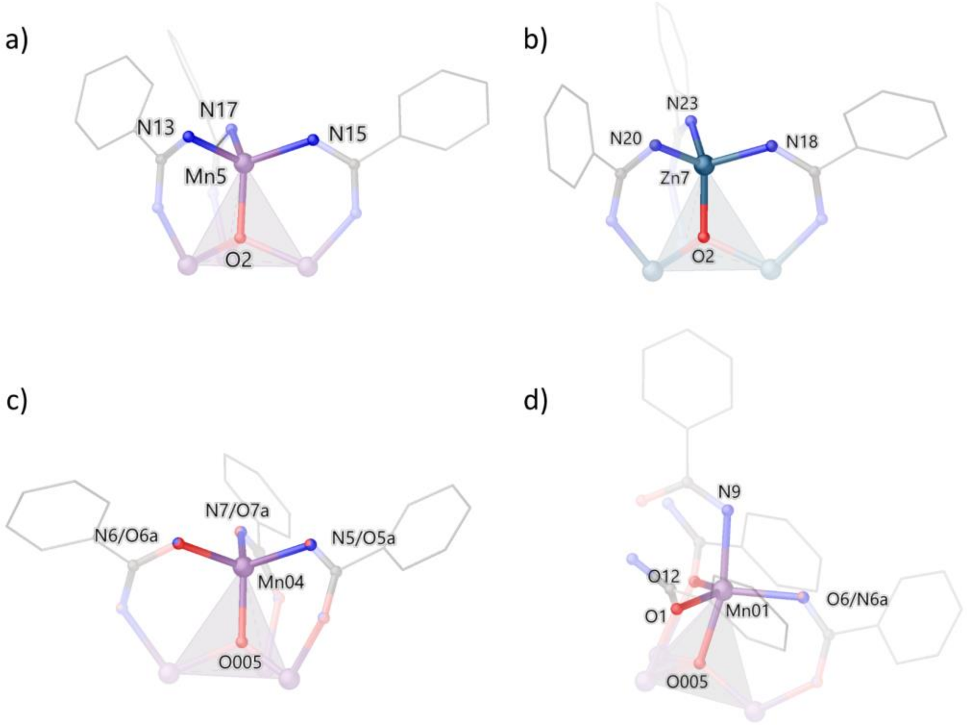

Figure S4. Representative metal coordination sphere geometries in **1-Mn** (a), **1-Zn** (b), and **2_2** (c,d).

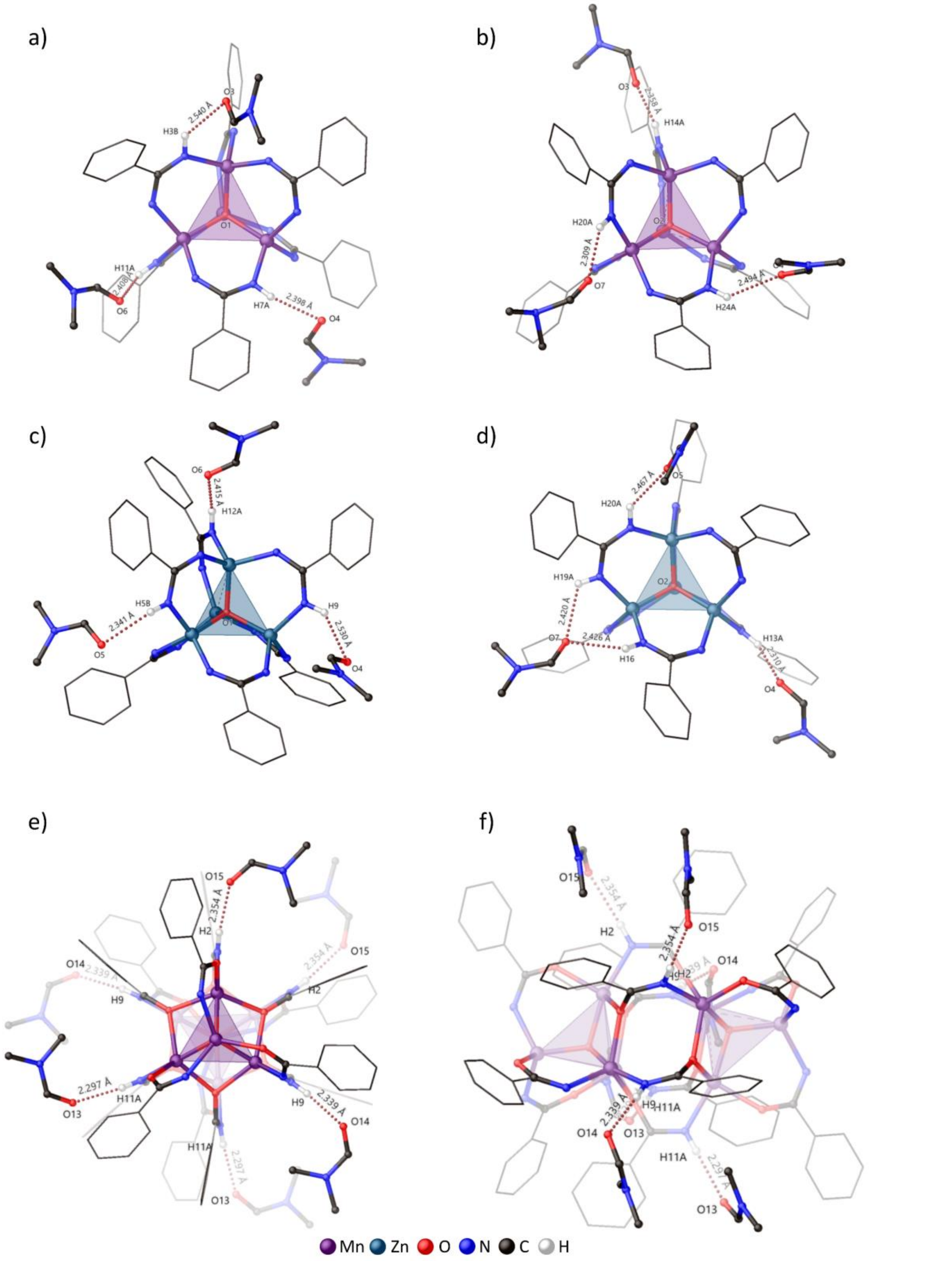

Figure S5. Analysis of the H-bonded DMF-solvated clusters in **1-Mn** (a,b: two crystallographically independent species), **1-Zn** (c,d: two crystallographically independent species), and **2₂** (e, f: along and side view on the same cluster)

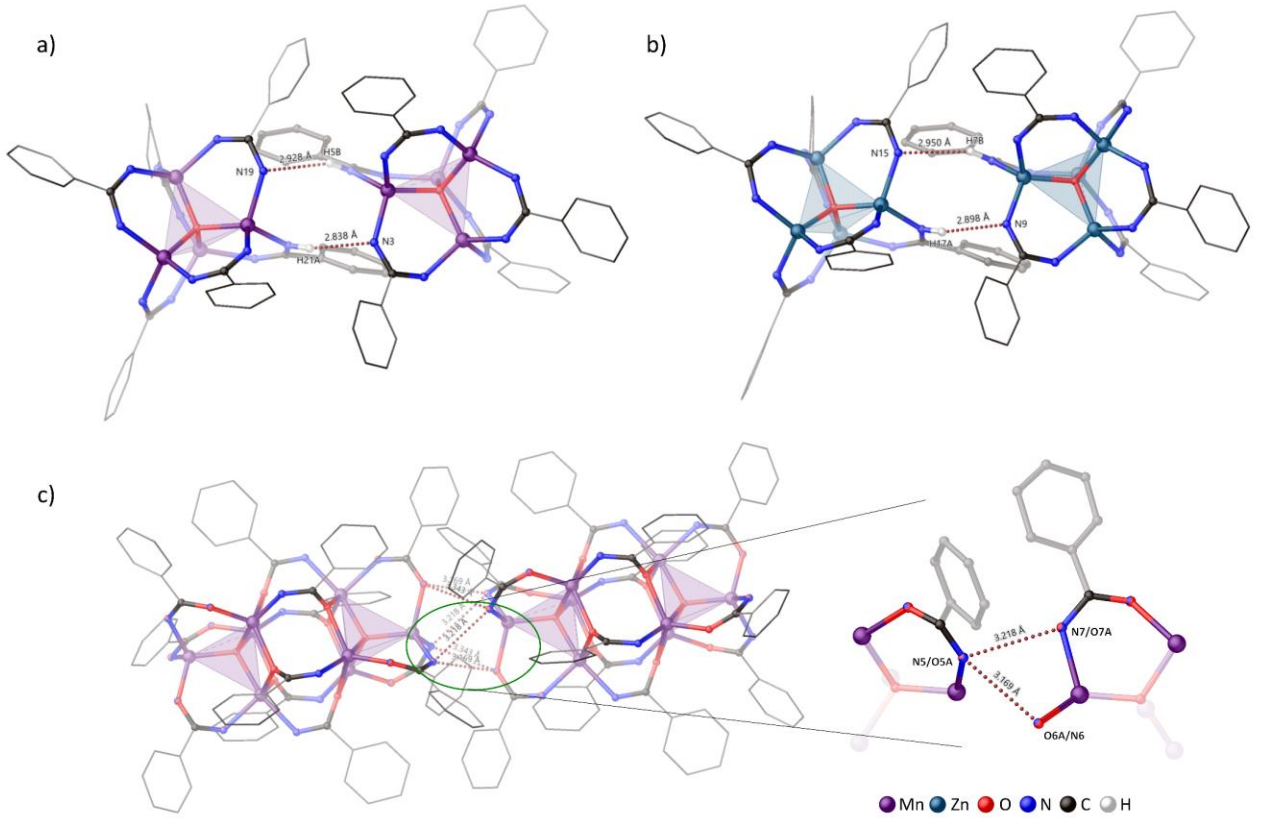

Figure S6. Analysis of the intercluster H-bonded bridges in **1-Mn** (a), **1-Zn** (b), and $\mathbf{2_2}$ (c)

2. FTIR Spectra

Due to the high sensitivity to air of clusters **1-Mn** and **2$_2$**, their FTIR-ATR characterization was performed under a protective layer of Nujol. The FTIR spectra of all compounds indicate the presence of amidinate anions. Specifically, bands at 3354, 3365, and 3340 cm^{-1}, and 3052, 3056, and 3052 cm^{-1} in the spectra of **1-Mn, 1-Zn,** and **2$_2$**, respectively, can be attributed to the stretching vibrations of amidinate N–H and aromatic C–H bonds. The bands at 1587 and 1466 for **1-Mn** and 1594 and 1472 cm^{-1} for **1-Zn** are likely attributed to the symmetric and asymmetric stretching of C=N bonds, respectively. In turn, in the spectrum of **2$_2$**, the band at 1590 cm^{-1} may be associated with the symmetric stretching of C=O bonds from benzamidate ligands, while bands at 1436 and 1410 cm^{-1} may correspond to the asymmetric stretching of C–N bonds in ligands adopting various coordination modes. The manganese clusters **1-Mn** and **2$_2$** show bands at 465 and 460 cm^{-1}, characteristic of Mn–O vibrations in the [(μ_4-O)M$_4$] motif. Cluster **1-Zn** shows a similar band for Zn–O stretching at 491 cm^{-1}. All samples exhibit bands around 2925, 2853, 1663, 1386, 1090, and 658 cm^{-1}, indicating the presence of DMF in the crystal lattice.

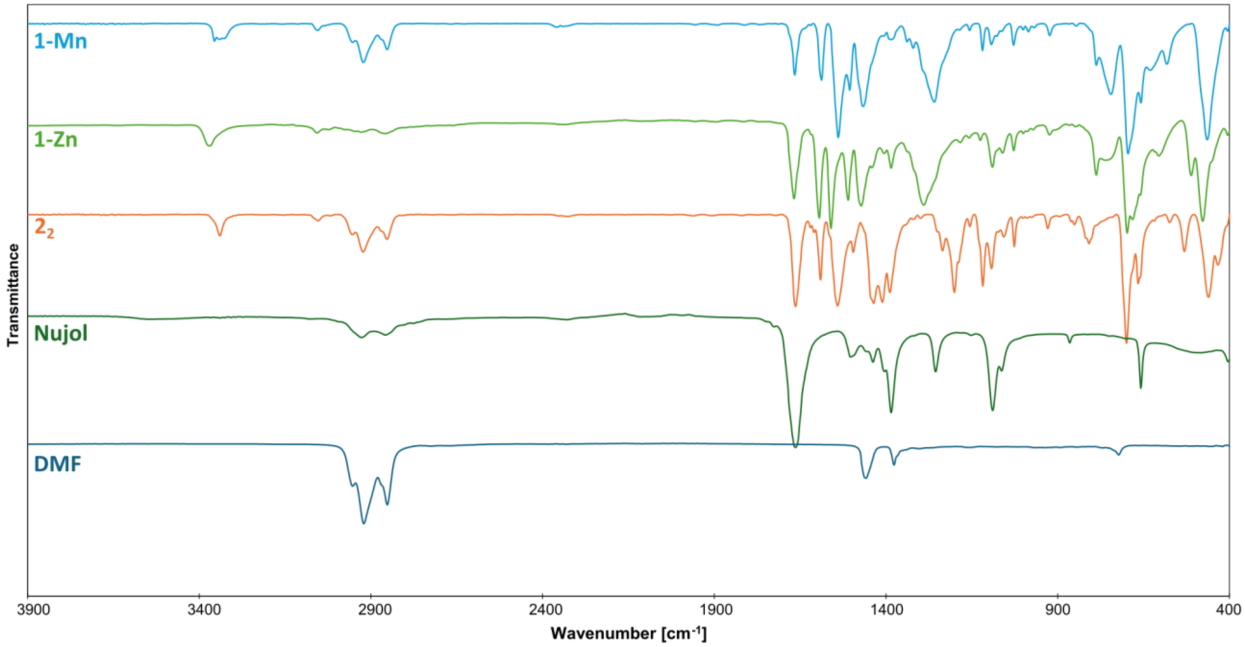

Figure S7. FTIR spectra of **1-Mn** (under a protective layer of Nujol), **1-Zn**, **2$_2$** (under a protective layer of Nujol), Nujol, and DMF.

3. NMR Spectra

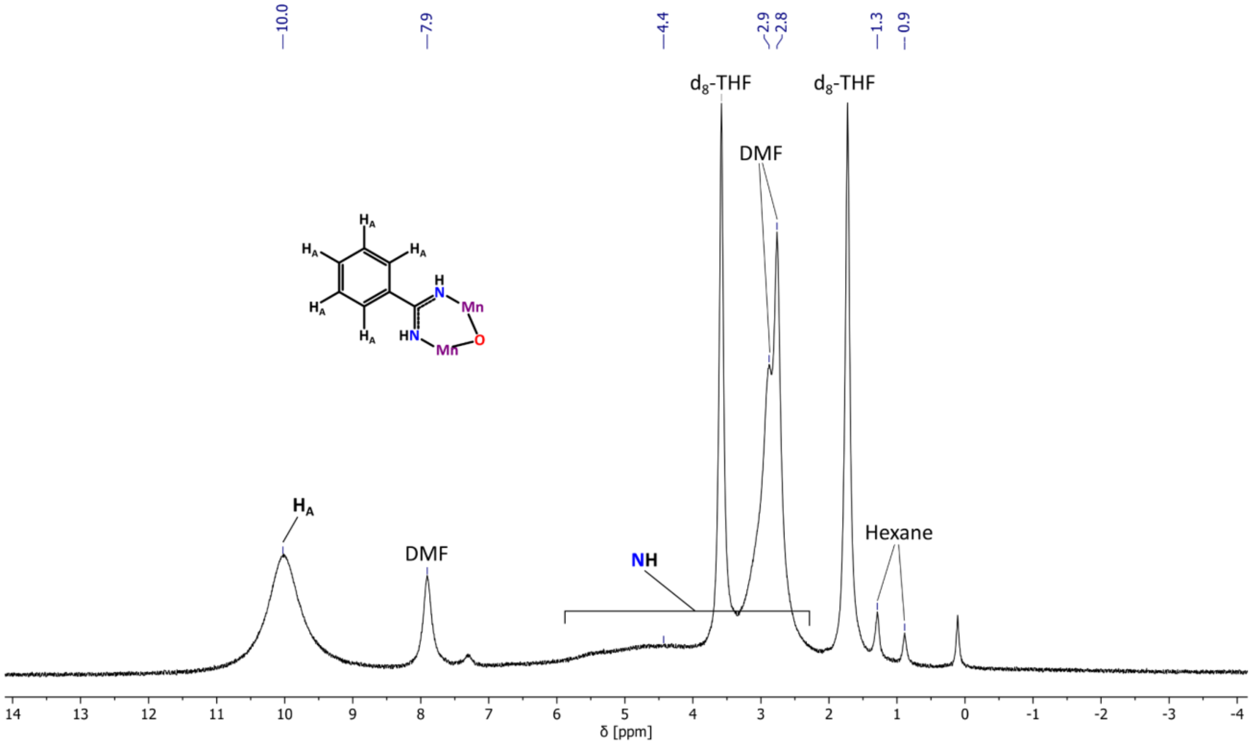

Figure S8. ^{1}H NMR spectrum of **1-Mn** in d_8-THF.

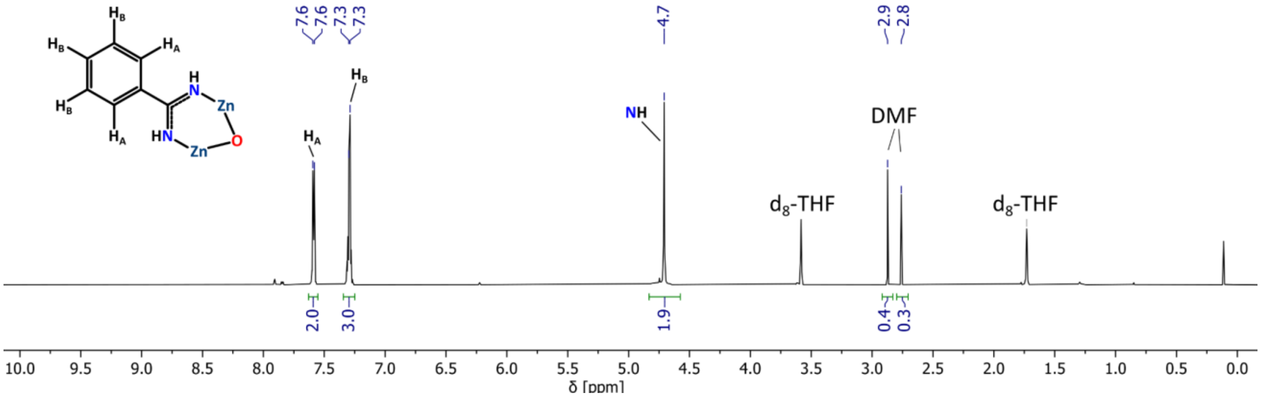

Figure S9. ^{1}H NMR spectrum of **1-Zn** in d_8-THF.

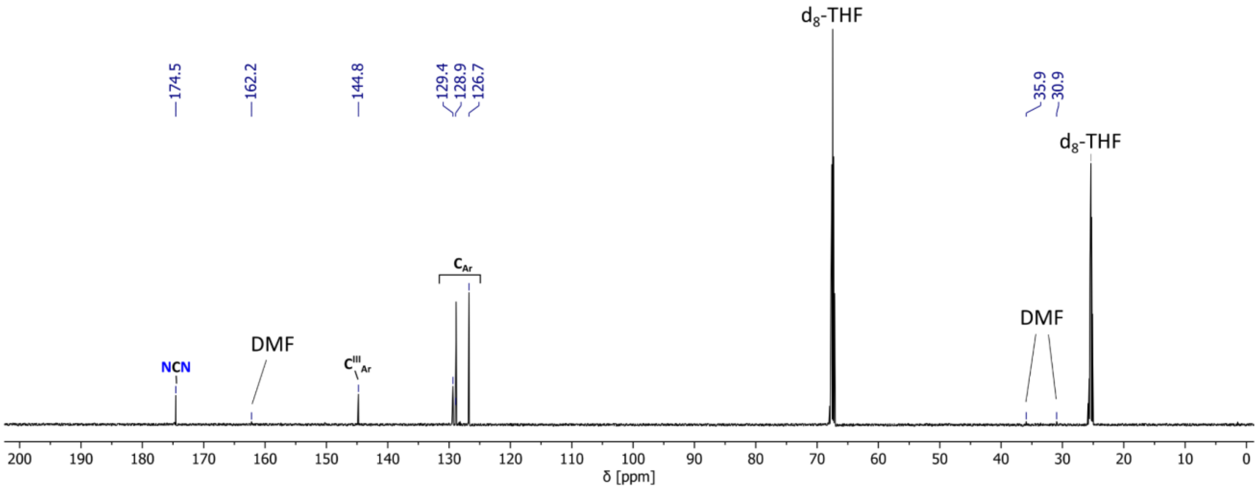

Figure S10. ^{13}C NMR spectrum of **1-Zn** in d_8-THF.

4. PXRD analysis

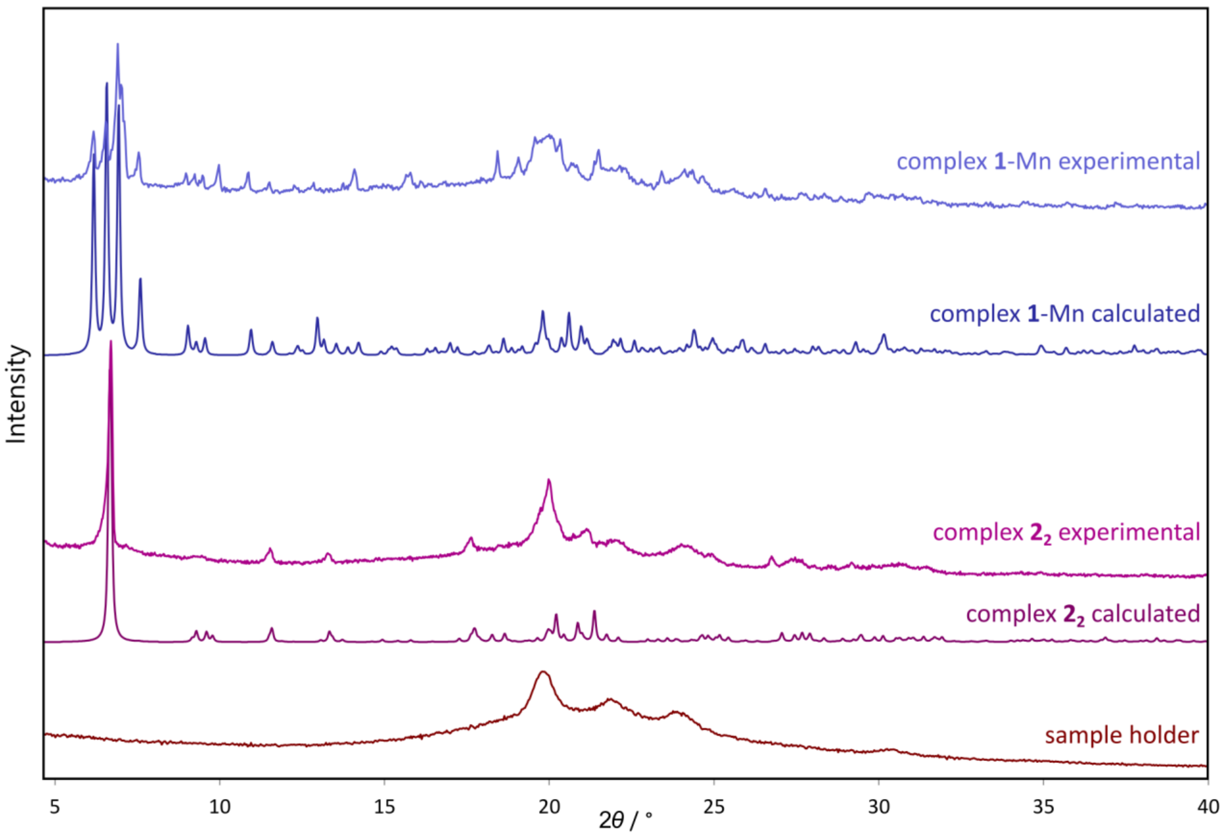

Figure S11. Experimental and calculated powder patterns of complexes **1-Mn** and **2**$_2$, as well as experimental powder pattern of the sample holder used during the experiments.

REFERENCES

(1) Terlecki, M.; Justyniak, I.; Leszczyński, M. K.; Lewiński, J. Effect of the Proximal Secondary Sphere on the Self-Assembly of Tetrahedral Zinc-Oxo Clusters. *Commun. Chem.* **2021**, *4* (1), 133 DOI: 10.1038/s42004-021-00574-3.

(2) Pinsky, M.; Avnir, D. Continuous Symmetry Measures. 5. The Classical Polyhedra. *Inorg. Chem.* **1998**, *37* (21), 5575–5582 DOI: 10.1021/ic9804925.

(3) Cotton, F. A.; Daniels, L. M.; Falvello, L. R.; Matonic, J. H.; Murillo, C. A.; Wang, X.; Zhou, H. Transition Metal (Mn, Co) and Zinc Formamidinate Compounds Having the Basic Beryllium Acetate Structure, and Unique Isomeric Iron Compounds. *Inorganica Chim. Acta* **1997**, *266* (1), 91–102 DOI: 10.1016/S0020-1693(97)05538-2.

(4) Cotton, F. A.; Daniels, L. M.; Jordan, G. T.; Murillo, C. A.; Pascual, I. Structural Variations in the Ligands around a Simple Oxo-Centered Building Block, the Tetrahedral $[M_4O]^{6+}$ Unit, M=Mn and Fe. *Inorganica Chim. Acta* **2000**, *297* (1–2), 6–10 DOI: 10.1016/S0020-1693(99)00252-2.

(5) Stokes, F. A.; Kloo, L.; Harford, P. J.; Peel, A. J.; Less, R. J.; Wheatley, A. E. H.; Wright, D. S. Towards the Synthesis of Guanidinate- and Amidinate-Bridged Dimers of Mn and Ni. *Aust. J. Chem.* **2014**, *67* (7), 1081–1087 DOI: 10.1071/CH14271.

(6) Krupiński, P.; Terlecki, M.; Kornowicz, A.; Justyniak, I.; Prochowicz, D.; van Leusen, J.; Kögerler, P.; Lewiński, J. Tetrahedral $M_4(\mu_4\text{-}O)$ Motifs Beyond Zn: Efficient One-Pot Synthesis of Oxido–Amidate Clusters via a Transmetalation/Hydrolysis Approach. *Inorg. Chem.* **2022**, *61* (20), 7869–7877 DOI: 10.1021/acs.inorgchem.2c00456.
